# RNA-binding proteome dynamics following ischemia-reperfusion injury in the kidney

**DOI:** 10.1016/j.isci.2026.118143

**Published:** 2026-11-07

**Authors:** Steffen Hinrichs, Katrin Bohl, Sophie Haselhorst, Francisco Lopes, Bernhard Wagener, Heike Göbel, Stefan Müller, Simon-Richard Finke, Tobias Neumann, Berit Pollmann, Constantin Rieger, Pia Rademann, Thomas Benzing, Peter Boor, Bernhard Schermer, Roman-Ulrich Müller, Michael Ignarski

**Affiliations:** 1 Department II of Internal Medicine and Center for Molecular Medicine Cologne, University of Cologne, Faculty of Medicine and University Hospital Cologne, Cologne, Germany; 2 CECAD, University of Cologne, Faculty of Medicine and University Hospital Cologne, Cologne, Germany; 3 Institute of Pathology, University of Cologne, Faculty of Medicine and University Hospital Cologne, Cologne, Germany; 4 Department of Anesthesiology and Intensive Care Medicine, University of Cologne, Faculty of Medicine and University Hospital Cologne, Cologne, Germany; 5 Department of Urology, Urologic Oncology, Robot-Assisted and Specialized Urologic Surgery, University of Cologne, Cologne, Germany; 6 Experimental Medicine, University of Cologne, Faculty of Medicine and University Hospital of Cologne, Cologne, Germany; 7 Institute of Pathology, University Hospital Aachen, RWTH Aachen University, Aachen, Germany; 8 Department of Nephrology, Medical Faculty, University Hospital Düsseldorf, Heinrich-Heine-University Düsseldorf, Düsseldorf, Germany; 9 Center of Excellence in Nephrology, University Hospitals Aachen, Cologne, Düsseldorf, North Rhine-Westphalia, Germany

**Keywords:** RNA-binding proteins, acute kidney injury, RNA interactome capture, RNA-protein interaction, metabolic enzymes

## Abstract

Acute kidney injury (AKI) is a frequent complication in hospitalized patients and is associated with increased mortality. Despite its relevance, no targeted therapies are available. RNA-binding proteins (RBPs) are essential regulators of cellular function and have been implicated in AKI, but a comprehensive analysis of their role is lacking. We investigated RBP regulation in porcine kidneys subjected to ischemia-reperfusion injury following cardiopulmonary resuscitation. To assess evolutionary conservation, RBP profiles were also obtained from human kidney tissue. After ischemia-reperfusion injury, we found RBPs exhibiting altered RNA-binding capacity, including metabolic enzymes previously implicated in ischemic stress. These findings suggest that energy metabolism is linked to RNA regulation during kidney injury. Renal ischemia-reperfusion injury alters the RNA-binding landscape, affecting both regulatory and metabolic RBPs. The conservation of these proteins in human kidneys highlights their translational relevance and identifies RNA-protein interactions as potential therapeutic targets in AKI.

## Highlights

• RNA interactome profiling reveals dynamic RBP remodeling following kidney IRI
• Translation-associated RBPs show increased RNA binding following IRI
• Metabolic enzymes emerge as dynamically regulated RNA-binding proteins
• Comparative analyses reveal conserved and species-specific features of the renal RBPome

## Graphical abstract

**Figure:**
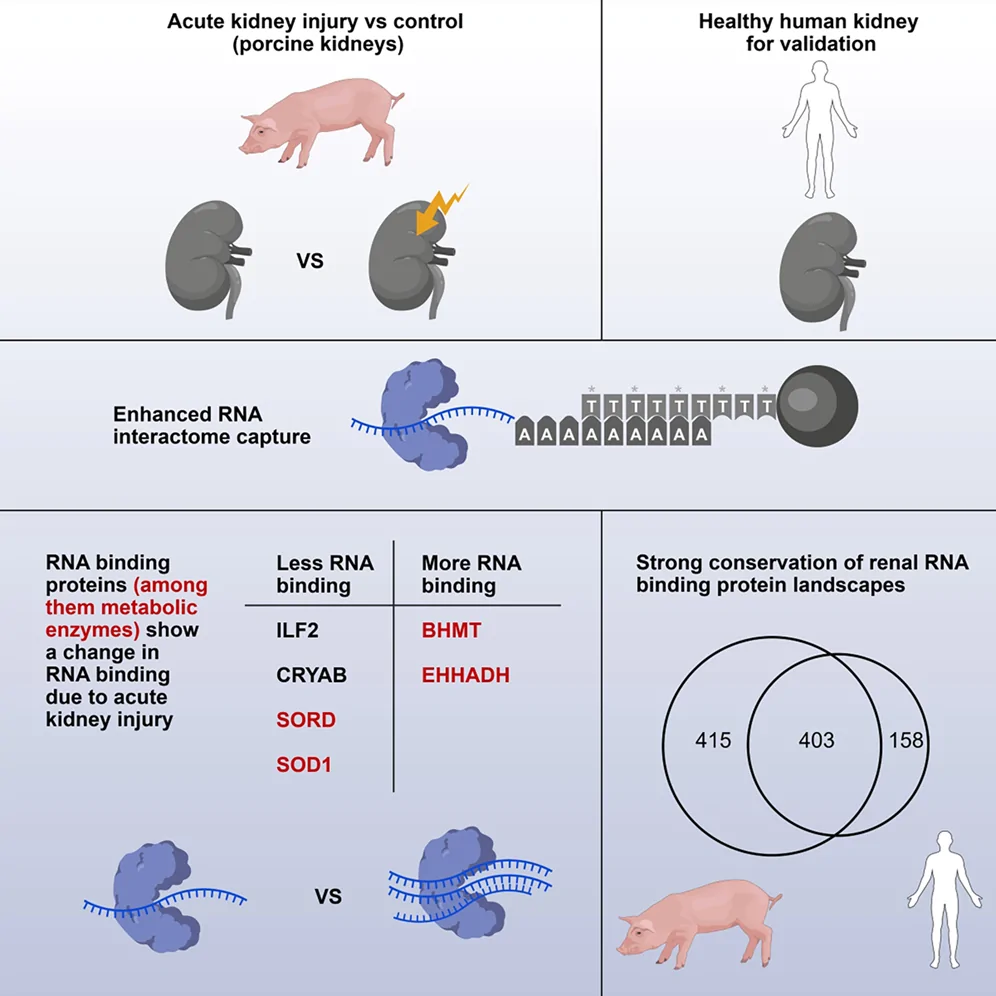

## Teaser

Biological sciences; Medical biochemistry; Biological database; Systems biology

## Introduction

Ischemia-reperfusion injury (IRI) occurs when the blood flow to an organ is restored after a period of complete or near-complete interruption. Although reperfusion is essential for tissue survival, it paradoxically triggers cellular dysfunction and death.^1^ In humans, renal IRI can result from conditions, such as cardiac arrest, shock, partial nephrectomy, or kidney transplantation, often leading to acute kidney injury (AKI).^1^ AKI itself is an independent risk factor for chronic kidney disease (CKD) and ultimately kidney failure.^1^^,^^2^^,^^3^^,^^4^ Despite the high prevalence and associated mortality, no targeted therapy currently exists.^5^^,^^6^^,^^7^ Therefore, a comprehensive understanding of the molecular mechanisms underlying IRI is critical for developing effective treatments for both IRI and AKI.

IRI involves a complex interplay of pathophysiological processes, including the generation of reactive oxygen species (ROS), inflammatory responses, and apoptosis.^1^ These events lead to profound metabolic reprogramming within renal tubular epithelial cells, altering lipid, glucose, and amino acid metabolism.^8^^,^^9^ Following renal IRI, a rapid and coordinated cellular response is essential, particularly within tubular epithelial cells and vascular endothelial cells, the principal cell types affected by ischemic and reperfusion stress.^1^

Recently, RNA-protein interactions have gained increasing attention as a fundamental regulatory mechanism in cell biology. RNA-binding proteins (RBPs) interact with RNA to mediate diverse aspects of post-transcriptional gene regulation, including mRNA processing, stability, translation, and modification.^10^^,^^11^ Conversely, enzymes such as kinases, ligases, ubiquitin proteases, and metabolic enzymes can be modulated by RNA, illustrating a bidirectional regulatory relationship between proteins and RNA.^10^^,^^12^^,^^13^^,^^14^^,^^15^^,^^16^ Furthermore, changes in RNA-binding affinity can dynamically alter RBP function and impact cellular homeostasis.^16^^,^^17^

Emerging evidence implicates RBPs in the pathophysiology of kidney disease, including a potential role in AKI.^10^ However, comprehensive RNA interactome datasets relevant to pathophysiologic conditions such as hypoxia, a central driver of AKI, remain scarce, with existing studies largely restricted to cultured cells and *Caenorhabditis elegans*.^12^^,^^18^ Notably, hypoxia has been shown to modulate the RNA interactome of renal epithelial cells, with two glycolytic enzymes identified as non-canonical RBPs exhibiting increased RNA-binding activity. These findings reinforce the concept that metabolic enzymes can function as non-canonical RBPs, linking metabolic regulation to RNA-mediated control.^12^^,^^16^^,^^19^

In parallel, recent advances in xenotransplantation have underscored the value of pigs as translational models for human kidney disease.^20^ The similarity of porcine anatomy, physiology, and immune responses to those of humans, particularly in relation to kidney structure and injury patterns, offers distinct advantages over rodent models for studying renal pathophysiology, including AKI.^21^^,^^22^^,^^23^^,^^24^

In this study, we aimed to make the essential step extending efforts to characterize renal RBPs to kidney tissue in a context relevant to human disease. This approach was chosen to deepen our understanding of AKI pathophysiology by characterizing RNA-protein interactions in porcine kidneys subjected to IRI following induced cardiac arrest. First, we confirmed that cardiac arrest followed by cardiopulmonary resuscitation successfully induced IRI in pigs, establishing this system as a valid model of renal IRI. Next, we applied enhanced RNA interactome capture (eRIC) to profile the baseline RBP content in healthy porcine and human kidney tissue and compared these datasets with published mouse kidney eRIC data to assess cross-species conservation and translational relevance. Finally, by integrating eRIC with proteomic and transcriptomic analyses in the porcine model, we identified RBPs that were either dysregulated or exhibited altered RNA-binding properties in response to IRI.

## Results

### Characterization of porcine cardiopulmonary resuscitation as a model for AKI

We aimed to obtain tissue from a model showing mild IRI-associated damage at the time point of sampling to examine the modulation of RBPs by IRI itself rather than downstream consequences of massive cell death. The average time to return of spontaneous circulation after cardiac arrest was 17.6 min, resulting in exactly this mild IRI phenotype. To characterize the model, we analyzed kidneys from pigs subjected to cardiopulmonary resuscitation after induction of cardiac arrest, as well as kidneys from control pigs for signs of IRI-induced renal damage using a multilayered approach, 5 h after resuscitation or after a corresponding observation period (Figure 1A). Omics datasets obtained included kidney proteome, transcriptome, and eRIC-based RNA interactome as well as the urinary proteome. Resuscitated pigs showed a mild but significant increase in serum creatinine and urea levels compared to control animals (Figures 1B and 1C). Blinded histopathology analysis by two independent nephropathologists revealed more severe signs of tubular damage in the IRI group, consistent with IRI-induced acute injury. This particularly included tubular vacuolization, epithelial flattening, defects of brush border, and tubules dilatation (Figure S1). We also analyzed the urinary proteome for known markers of AKI. This approach revealed significant increases in urinary alpha-1-microglobulin, cystatin C, L-FABP, NGAL, and TIMP1 (Figure 1D; Table S1).

**Figure 1:**
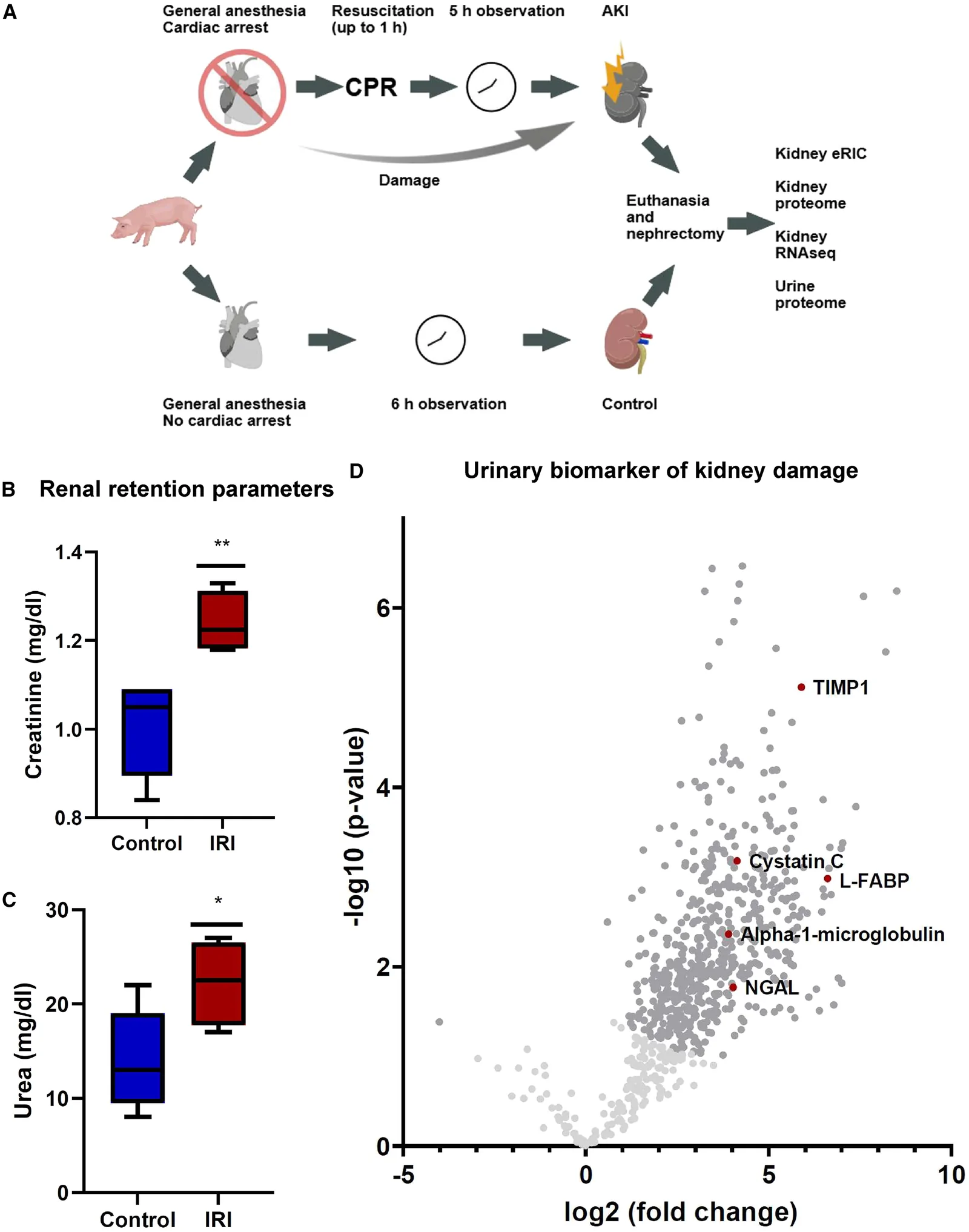
The porcine cardiac arrest model results in an acute kidney injury (AKI) phenotype (A) The cardiopulmonary resuscitation (CPR) scheme illustrates the workflow of the porcine ischemia-reperfusion injury (IRI) model and the corresponding omics datasets. In the IRI group (upper image), cardiac arrest was induced using alternating current and maintained for 10 min before automated resuscitation. Pigs that achieved return of spontaneous circulation were stabilized and monitored for 5 h, during which their kidneys experienced ischemia-reperfusion injury resulting in AKI. Animals were then euthanized and kidneys were collected. In the control group (lower image), no cardiac arrest was induced; pigs were monitored for the same duration before euthanasia and kidney collection. Control kidneys were free of IRI-related damage. Created with BioGDP.com.^25^ (B) Boxplot showing serum creatinine levels (mg/dL) in the IRI and control groups. Statistical significance was determined by *t* test (∗∗*p* < 0.01). The IRI group comprised four samples (one per animal), and the control group comprised five samples (one per animal), three of which were collected prior to induction of cardiac arrest as additional controls. Statistical significance was calculated with an unpaired *t* test (GraphPad Prism v.9.3.1, GraphPad Software, Boston, Massachusetts USA, www.graphpad.com). The whiskers indicate the minimum and maximum value, the box extends from the 25th to 75th percentiles, and the line in the box represents the median. (C) Boxplot showing serum urea levels (mg/dL) in the IRI and control groups. Statistical significance was determined by *t* test (∗ = *p* < 0.05). Samples as in (B). Statistical significance was calculated with an unpaired *t* test (GraphPad Prism v.9.3.1, GraphPad Software, Boston, Massachusetts USA, www.graphpad.com). The whiskers indicate the minimum and maximum value, the box extends from the 25th to 75th percentiles, and the line in the box represents the median. (D) Volcano plot showing the *t* test comparison of the urinary protein intensities between the IRI and control groups. The x axis indicates the log_2_ fold change in abundance, and the y axis represents the -log_10_*p* value. The Student’s *t* test was performed with a S_0_ = 0.2. AKI markers are highlighted in red, significant proteins (*q* value <0.05) as gray and not significant light gray. Samples as in (B) and (C). The corresponding dataset is provided in Table S1.

Proteomic analysis of porcine kidney tissue identified 3,239 proteins, 171 of which showed significant changes in abundance following IRI (Table S2). Principal component analysis (PCA) revealed a clear separation between control and IRI groups, indicating distinct proteomic profiles associated with the intervention (Figure S2). Complementary transcriptome analysis detected 15,722 transcripts, with 4,947 significantly dysregulated in response to IRI (Table S3). The PCA of transcriptomic data similarly showed distinct clustering of samples by experimental condition (Figure 2A). Gene Ontology (GO) and pathway enrichment analyses of both datasets highlighted pronounced alterations in immune-related processes, apoptosis signaling pathways, and metabolic functions (Figure 2B; Tables S4, S5, S6, S8, and S9).

**Figure 2:**
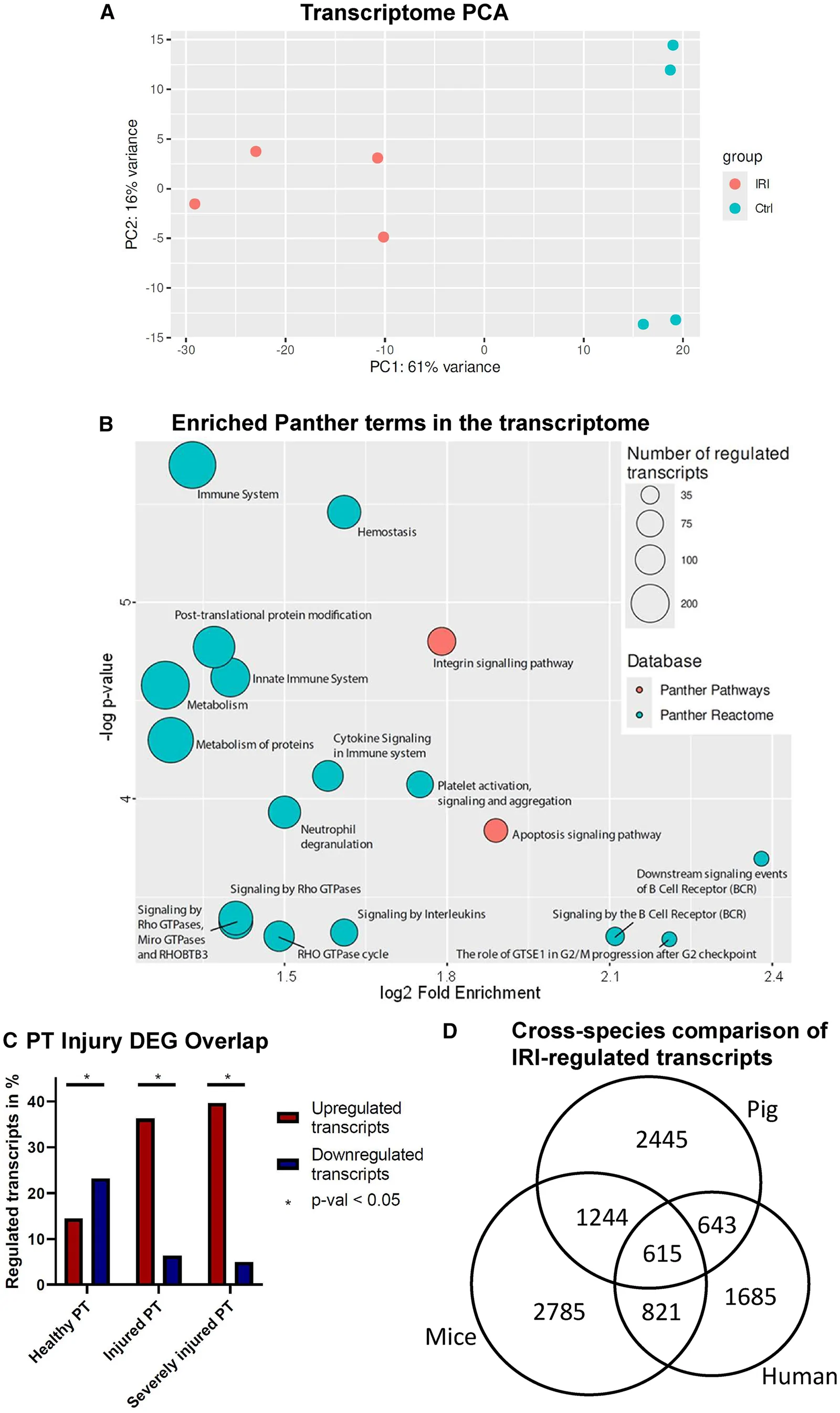
Analysis of porcine kidney transcriptome after ischemia-reperfusion injury (A) Principal component analysis of the kidney transcriptome showing clear separation along the first component between the ischemia-reperfusion injury (IRI) group (red, four samples from four animals) and the control group (blue, four samples from two animals) based on normalized read counts. (B) Bubble plot showing significantly enriched PANTHER pathway (red) and PANTHER Reactome (blue) terms identified by Fisher’s exact test (FDR <0.05) for differentially regulated porcine kidney transcripts between the IRI and control groups. The *x* axis shows the fold enrichment (log_2_), and the *y* axis shows the *p* value (−log_10_). Bubble size represents the number of transcripts annotated to each pathway. Samples as in (A). Pathways with fold enrichment <1 are not displayed. Complete pathway lists are provided in Tables S8 and S9. (C) Bar chart showing the percentage of upregulated (red) or downregulated (blue) porcine transcripts overlapping with differentially expressed genes (DEGs) from healthy, injured, and severely injured proximal tubule (PT) cells in a single-nucleus RNA-seq dataset.^26^ The *y* axis indicates the proportion of regulated DEGs within each PT state relative to all DEGs in that state. Statistical significance between up- and downregulated DEGs was determined using Fisher’s exact test (∗ = *p* < 0.05), see Table S11. (D) Venn diagram showing a cross-species overlap of significantly regulated transcripts between the porcine transcriptome dataset, a murine IRI dataset,^27^ and a human IRI dataset.^28^ Detailed gene lists are provided in Table S12.

Analysis of the pig transcriptome for expression of marker genes associated with healthy and injured proximal tubule (PT) segments (S1, S2, and S3), as described in published mouse single-nucleus RNA-sequencing (snRNA-seq) data,^26^ revealed clear evidence of tubular injury and loss of healthy PT cells. Marker genes characteristic of healthy PT segments were predominantly downregulated, whereas those injured and severely injured PT were predominantly upregulated in the IRI group (Figure 2C; Tables S10 and S11).

Furthermore, approximately half of the transcripts dysregulated in the porcine kidney (2,502/4,947) overlapped with genes previously reported as regulated in mouse and/or human (IRI) datasets (Figure 2D; Table S12).^27^^,^^28^ Among the transcripts shared across all three species, the most enriched PANTHER pathways included integrin signaling, angiogenesis, inflammatory, and apoptosis signaling, Ras pathway, and interleukin signaling (Figure S3; Table S13). Collectively, these findings demonstrate that the cardiac arrest and cardiopulmonary resuscitation pig model reproduces the characteristic molecular signature of renal IRI, closely mirroring damage-associated responses observed in both mouse models and human kidney injury.

### RNA interactomes of human and pig kidney show a high degree of evolutionary conservation underlining translational relevance

After validating the porcine cardiac arrest model as a suitable system for mapping IRI-induced changes of the RBP landscape, we continued with the global characterization of the RNA-binding proteome (RBPome). Using eRIC, we sought to define the RBP landscape in porcine and human kidney tissue.^19^ For this, kidney slices were UV crosslinked, a method known to induce covalent bond formation between proteins and RNA only when they are in direct contact.^14^ Subsequently, polyadenylated RNA was isolated from the crosslinked samples, with covalently bound RBPs retained for mass spectrometry. Non-crosslinked samples served as negative controls, as RBPs not covalently attached to RNA were removed during purification, leaving only nonspecific binding proteins.^14^^,^^19^ Enrichment of a protein in the crosslinked versus non-crosslinked samples indicated direct RNA binding.^12^^,^^14^^,^^18^^,^^19^^,^^29^ Application of the eRIC protocol to both species revealed robust enrichment of RBPs in the UV-C-crosslinked samples compared to the non-irradiated samples (Figure 3A). The PCA of the mass spectrometry data further demonstrated a clear separation between crosslinked and non-crosslinked samples in both pig and human kidneys (Figures S4 and S5), confirming the specificity and reproducibility of RBP capture.

**Figure 3:**
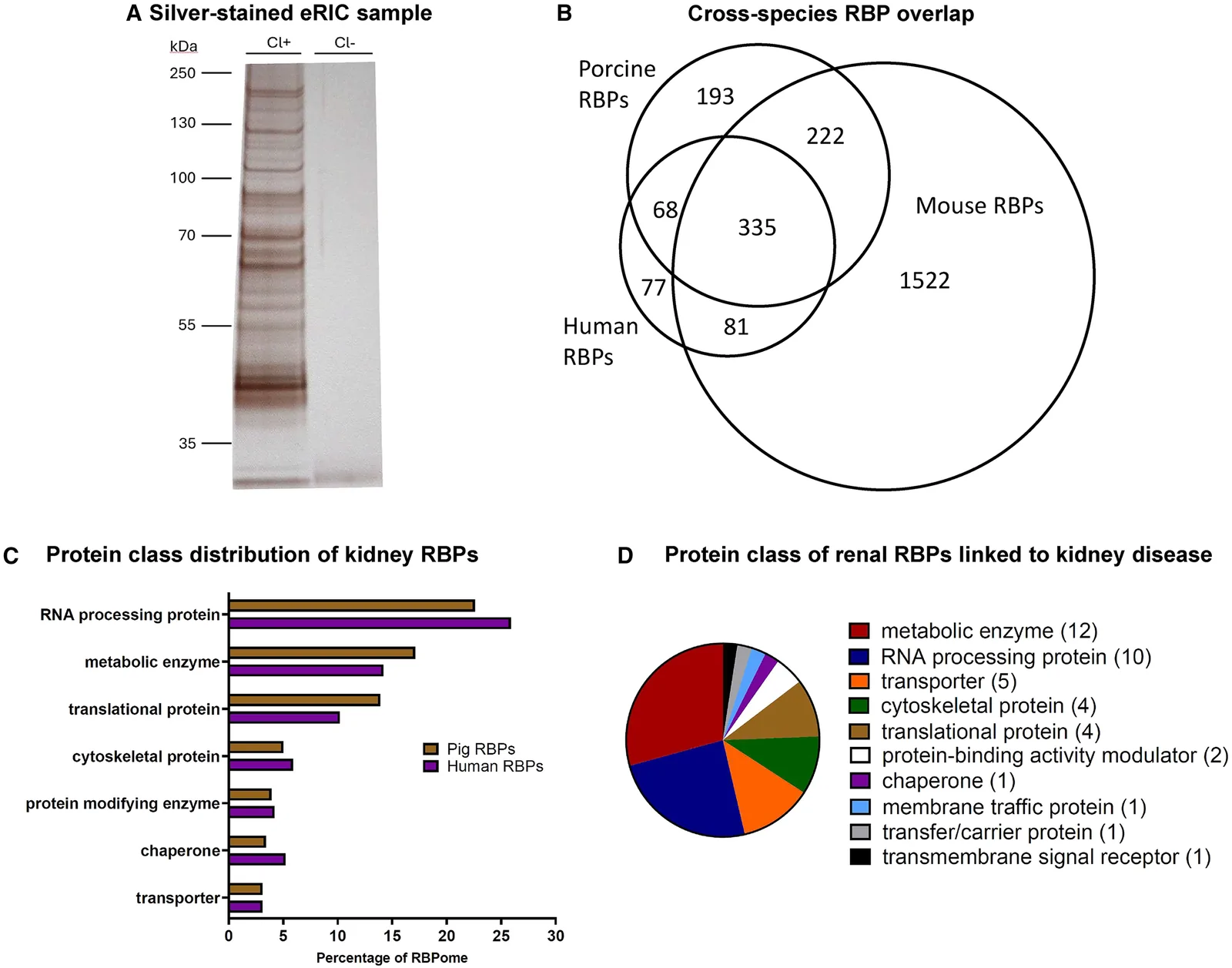
Characterization and inter-species comparison of kidney RNA interactomes reveals strong similarities between pig and human (A) Silver-stained polyacrylamide gel showing enrichment of porcine kidney RNA-binding proteins (RBPs) in crosslinked (Cl+) but not in non-crosslinked (Cl−) enhanced RNA interactome capture (eRIC) samples. Molecular weight marker indicated in kDa. (B) Venn diagram showing the overlap of RBPs (unique gene names) between the pig and human eRIC datasets generated in this study and the published mouse kidney eRIC dataset.^19^ Corresponding gene lists are provided in Table S19. (C) Bar chart showing the seven most represented PANTHER protein classes in porcine (brown) and human (purple) kidney eRIC datasets. The *x* axis indicates the percentage of each class within the full eRIC dataset. Proteins without PANTHER class assignment were excluded. The “RNA metabolism protein” category was renamed “RNA processing protein”, and “metabolite interconversion enzyme” was renamed “metabolic enzymes”. Complete protein class assignments are provided in Table S20. (D) Pie chart showing PANTHER protein class annotations for RBPs associated with kidney disease identified in pig and/or human kidney eRIC datasets. These proteins have been previously implicated in kidney pathophysiology^10^ or are listed as high-evidence disease-associated genes in the Kidney-Genetics Documentation database.^30^ Proteins without PANTHER class assignments were excluded. The “RNA metabolism protein” and “metabolite interconversion enzyme” categories were renamed as in (C). The corresponding RBP list is provided in Table S22.

To ensure high confidence in RBP identification, we filtered the eRIC-derived proteomic data using a two-tier classification system. Proteins were categorized as class 1 if they were significantly enriched in crosslinked versus non-crosslinked samples (Student’s *t* test) or detected exclusively in crosslinked samples. Furthermore, class 2 did not meet class 1 criteria but had an RBP2GO human score greater than 20.^31^ Using this approach, we identified 818 RBPs in porcine kidneys (control and IRI) and 561 RBPs in human kidneys (Tables S14 and S15). Notably, this analysis uncovered 35 previously unrecognized RBPs in pigs and 17 in humans, expanding the known repertoire of kidney-associated RBPs.^31^^,^^32^^,^^33^^,^^34^^,^^35^

GO enrichment analysis of both the pig and human eRIC datasets revealed strong overlap and significant enrichment of molecular function terms associated with RNA binding, including poly(A) binding and mRNA binding, when compared with corresponding proteome datasets (Figures S6 and S7; Tables S16, S17, and S18). Comparison of our data with a published mouse kidney RBP dataset,^19^ identified 335 renal RBPs shared across all three species (Figure 3B; Table S19), highlighting a conserved core of RBPs in mammalian kidneys.

PANTHER protein class analysis revealed a high degree of similarity between porcine and human kidneys in the most abundant RBP classes, including RNA-processing proteins and metabolic enzymes (Figure 3C; Table S20).^36^^,^^37^^,^^38^ Notably, among the RBPs not previously associated with RNA binding, 15 of 35 porcine and 7 of 17 human RBPs were annotated as metabolic enzymes (Table S21).^36^^,^^37^^,^^38^ Several transport proteins were also identified as novel RBPs, including aquaporin-1 (AQP1) and large neutral amino acids transporter small subunit 2 (SLC7A8) in pigs, as well as voltage-dependent calcium channel gamma-2 subunit (CACNG2) and sodium-dependent phosphate transport protein 4 (SLC17A3) in human (Table S21), suggesting potential new roles in RNA regulation.^36^^,^^37^^,^^38^

To explore the potential relevance of the RBPs identified in kidney disease, we compared our porcine and human RBP datasets with high-confidence kidney disease-associated genes from the Kidney-Genetics Documentation Database,^30^ as well as RBPs previously implicated in kidney pathophysiology.^10^ This analysis identified 47 RBPs linked to kidney disease, encompassing both canonical RBPs and an enrichment of metabolic enzymes and transport proteins (Table 1; Figure 3D; Table S22), highlighting potential functional connections between RNA binding and renal pathology.

**Table 1:** Metabolic enzymes and transporter proteins identified as RBPs and their association with genetic disease

Gene name	Protein class	Disease association	Pig kidney RBP eRIC	Human kidney RBP eRIC	Mice kidney RBP eRIC
CA2	metabolic enzyme	osteopetrosis, with renal tubular acidosis	+	+	+
EHHADH	metabolic enzyme	Fanconi renotubular syndrome 3	+	+	+
ETFB	metabolic enzyme	glutaric acidemia IIB	+	+	+
FAH	metabolic enzyme	tyrosinemia, type I	+	–	+
FH	metabolic enzyme	leiomyomatosis and renal cell cancer | fumarase deficiency	+	–	+
GATM	metabolic enzyme	cerebral creatine deficiency syndrome 3 | Fanconi renotubular syndrome 1	+	+	–
GRHPR	metabolic enzyme	hyperoxaluria, primary, type II	+	–	–
HAAO	metabolic enzyme	vertebral, cardiac, renal, and limb defects syndrome 1	+	–	+
HOGA1	metabolic enzyme	hyperoxaluria, primary, type III	+	–	–
LDHA	metabolic enzyme	glycogen storage disease XI	+	+	+
PGK1	metabolic enzyme	phosphoglycerate kinase 1 deficiency	+	+	+
PHGDH	metabolic enzyme	Neu-Laxova syndrome 1| phosphoglycerate dehydrogenase deficiency	+	+	–
ATP1A1	transporter	(renal) hypomagnesemia, seizures, and impaired intellectual development 2 […] (OMIM^39^; Schlingmann et al;^40^)	+	+	+
SEC61A1	transporter	tubulointerstitial kidney disease 5	+	+	+
SLC12A1 (NKCC2)	transporter	Bartter syndrome, type 1	+	–	+
SLC4A4	transporter	renal tubular acidosis, proximal, with ocular abnormalities	+	–	+
XPO5	transporter	nephrotic syndrome (Braun et al.^41^)	+	–	–

RBPs listed in the Kidney-Genetics Documentation Database^30^ are annotated with Online Mendelian Inheritance in Man (OMIM)^39^ entries or other reported disease associations. The table includes gene name, PANTHER protein class,^36^^,^^37^^,^^38^ and detection in pig, human, or mouse^19^ kidney eRIC datasets. The protein class of fumarylacetoacetase was manually assigned based on gene ontology annotations.^33^^,^^34^^,^^35^ Full table is provided in Table S22.

In order to validate the RNA-binding capability of the identified metabolic enzymes, we used biotin-based visualization of RBP-coupled RNA.^42^ Four metabolic enzymes—fumarate hydratase (FH), enoyl-CoA hydratase and 3-hydroxyacyl-CoA dehydrogenase (EHHADH), betaine-homocysteine S-methyltransferase (BHMT), and sorbitol dehydrogenase (SORD)—were selected for validation because they displayed robust RNA-binding signals in our dataset, represent distinct metabolic pathways, and have not been widely established as canonical RBPs. Together, these enzymes provide a representative cross-section of the metabolically diverse RBPs identified in our study. In brief, FLAG-tagged metabolic enzymes were overexpressed in HEK293T cells, UV crosslinked, and lysed. RBP-RNA complexes were immunoprecipitated, followed by on-bead ligation of pCp-Biotin to the associated RNA and streptavidin-based detection. A fraction of the same samples was used to detect the respective RBP by anti-FLAG immunoblotting. To account for differences in the RNA-binding capacity of the individual proteins, low (40 U) and high (400 U) RNase I concentrations were applied. Non-crosslinked and IgG controls were included to assess the specificity of the RBP-RNA signal.^42^ All four selected metabolic enzymes revealed a specific biotin signal at the expected molecular weight, with strong enrichment in the UV-crosslinked samples. These results confirm that the selected metabolic enzymes physically interact with RNA (Figures S8 and S9).

### The impact of IRI on RBPs: Changes in expression and modulation of RNA-binding capacity

We next examined whether RBPs respond to IRI-induced stress and whether such responses occur at the level of protein expression or changes RNA-binding activity. Analysis of class 1 and 2 RBPs in the porcine kidney proteome revealed that six RBPs were significantly differentially expressed following IRI (*q* value <0.05) (Table S23). In contrast, RNA-seq of the same samples revealed more extensive IRI-induced changes at the transcriptome level, with 299 mRNAs significantly dysregulated (*q* value <0.05) (Table S23).

To investigate IRI-induced changes in RNA binding, we compared the porcine kidney eRIC dataset under IRI with controls, normalizing for protein abundance to account for potential expression-level changes. This analysis identified 27 RBPs with differential RNA binding. Notably, 23 RBPs displayed altered RNA binding in eRIC without corresponding changes in protein abundance, whereas four RBPs were significantly regulated at the protein level but showed no change in RNA binding (Figures 4A and 4B; Tables S23 and S24), highlighting distinct layers of post-transcriptional regulation in response to IRI.

**Figure 4:**
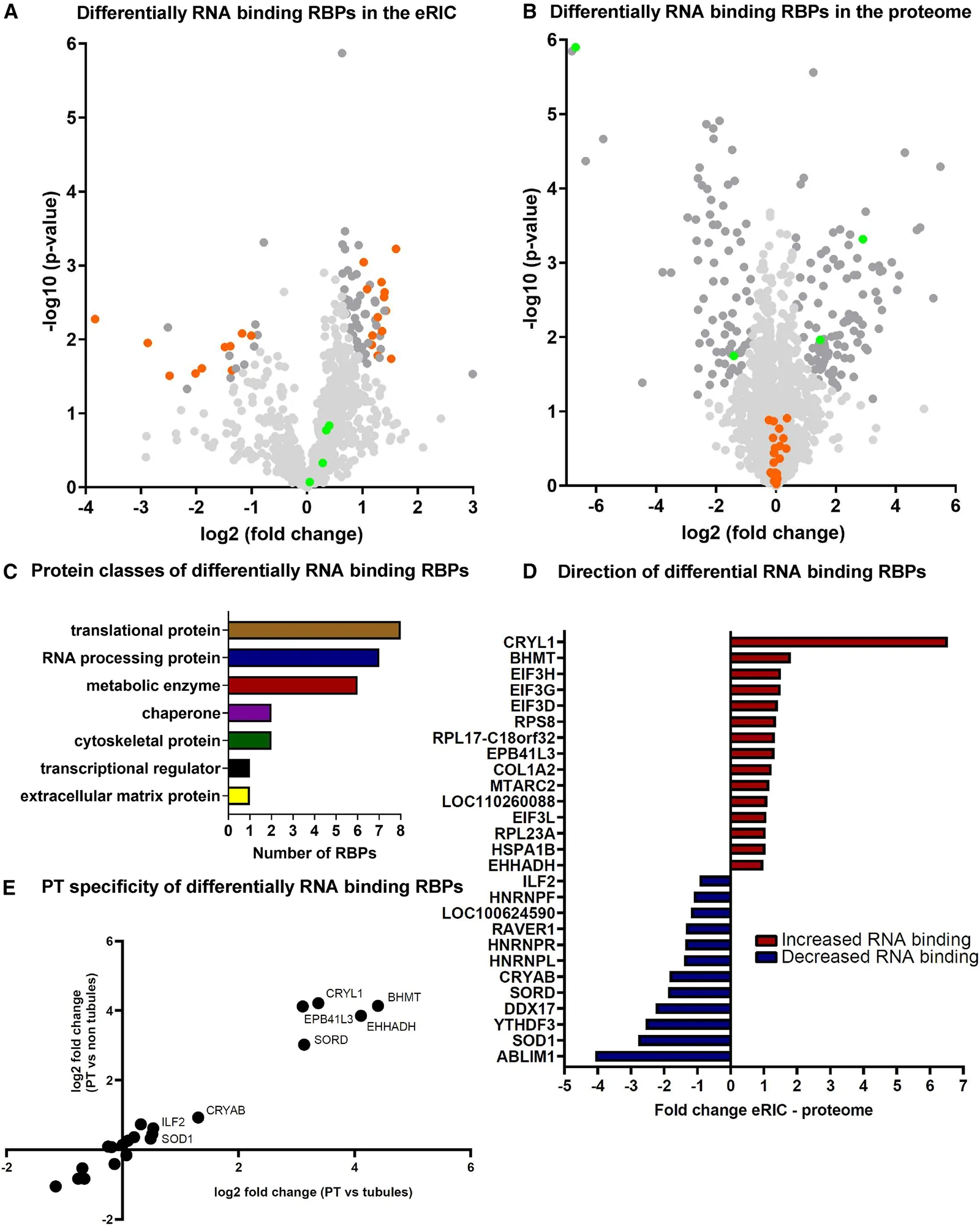
Analysis of differentially RNA binding RBPs reveals IRI-induced changes in the kidney RNA interactome (A and B) Volcano plots showing *t* test comparisons of protein LFQ intensities between IRI and control groups for enhanced RNA interactome capture (eRIC) (A) and proteome (B) datasets. The *x* axis represents the log fold change and the *y* axis shows –log_10_*p* value. Student’s *t* tests were performed with S_0_ = 0.2. RBPs differentially regulated in eRIC but not in the proteome are highlighted orange, while RBPs regulated in the proteome but not in eRIC are shown in green. Significantly regulated proteins (*q* value <0.05) are colored dark gray, and non-significant proteins are light gray. The IRI group included three samples, the control group included four samples from two animals. The corresponding datasets are provided in Tables S2 and S14. (C) Bar chart showing PANTHER protein classes of differentially RNA-binding RBPs. The *x* axis indicates the number of RBPs annotated to each protein class. Proteins without PANTHER class assignments were excluded. The “RNA metabolism protein” category was renamed “RNA processing protein”, “metabolite interconversion enzyme” was renamed “metabolic enzymes” and “gene-specific transcriptional regulator” was shortened to “transcriptional regulator”. The corresponding RBP list is provided in Table S24. (D) Bar chart showing changes in RNA binding of differential RBPs. The *x* axis represents the difference between log_2_ fold changes from the eRIC and proteome datasets ((log_2_ FC of eRIC IRI vs. control) – (log_2_ FC of proteome IRI vs. control)) data correspond to Student’s *t* test results for crosslinked eRIC samples versus control and for proteome samples. The corresponding RBP list is provided in Table S24. (E) Scatterplot showing differentially RNA-binding RBPs relative to their expression in proximal tubules (PT). The *x* axis represents the log_2_ fold change of PT versus other tubules, and the y axis represents the log_2_ fold change of PT versus non-tubule cells. PT specificity was calculated as log_2_(1/(ranking_PT/mean ranking of non-tubules or other tubules)), using ranking values derived from a human single nucleus RNA-seq dataset.^43^ Corresponding data are provided in Tables S24 and S25.

Using the PANTHER classification system and GO biological processes annotation, we found that proteins associated with translation represented the most abundant class among the differentially bound RBPs (Figure 4C; Table S24). All eight detected eukaryotic translation initiation factors—EIF3D, EIF3G, EIF3H, EIF3L, EIF3C (LOC110260088), RPL17-C18orf32, RPL23A, and RPS8—showed increased RNA binding upon IRI (Figure 4D; Table S24).

Seven of the 27 differentially bound RBPs were RNA-processing proteins—DDX17, HNRNPF, HNRNPL, HNRNPR, RNA helicase (LOC100624590), ribonucleoprotein PTB binding 1 (RAVER1), and YTHDF3—including four ribonucleoproteins, all of which showed decreased RNA binding (Figure 4D; Table S24). The third largest group comprised metabolic enzymes, including BHMT, CRYL1, EHHADH, MTARC2, SOD1, and SORD (Figure 4C).

Additionally, several other protein classes were differentially bound upon IRI: chaperones (CRYAB and HSPA1B), cytoskeletal proteins (ABLIM1 and EPB41L3), a gene-specific transcriptional regulator (ILF2), and an extracellular matrix protein (COL1A2) (Figure 4C; Table S24). Notably, 19 of the 27 RBPs were also detected as kidney RBPs in our human eRIC dataset and/or in the published mouse kidney eRIC data,^19^ highlighting their cross-species relevance (Table S24).

Considering that proximal tubules (PTs) are among the first cell types affected by IRI and AKI,^1^ we examined the expression of the differentially bound RBPs in human kidney snRNA-seq data.^43^ Specifically, we compared their expression levels in PT cells versus other kidney cell types to assess cell-type specificity. This analysis identified five RBPs—BHMT, CRYL1, EHHADH, EPB41L3, and SORD—with high specificity for PTs (Figure 4E; Tables S24 and S25). Notably, four of these five RBPs are well-characterized metabolic enzymes, representing a greater proportion of metabolic enzymes than observed in the overall set of differentially bound RBPs. Finally, to assess the potential relevance of the differentially bound RBPs to the pathophysiology of AKI, we performed a literature search for previous reports linking these RBPs to AKI or IRI/ischemia. We identified six RBPs with documented associations, four of which are metabolic enzymes (Table 2), further supporting their potential role in renal response to ischemic injury.

**Table 2:** Differentially RNA-bound RBPs associated with acute kidney injury

Gene name	Protein name	Protein class	Human kidney RBP	Mice kidney RBP	AKI association
BHMT	betaine-homocysteine S-methyltransferase	metabolic enzyme	+	+	AKI: involved in the metabolism of sulfur-containing amino acids. Restricting sulfur-containing amino acids results in protection from AKI in mouse (Koehler et al.^44^).
CRYAB	alpha-crystallin B chain	chaperone	–	–	ischemia: shown to have a cardioprotective effect against ischemia (Cubedo et al.^45^).
EHHADH	enoyl-CoA hydratase/peroxisomal bifunctional enzyme	metabolic enzyme	+	+	AKI: loss of EHHADH has been linked to male-specific proximal tubular injury in mice (Renea-Robles et al.^46^) and to accelerated tubulointerstitial injury in diabetic kidney disease (Kan et al.^47^).
ILF2	interleukin enhancer-binding factor 2	gene-specific transcriptional regulator	+	+	IRI: inhibits cardiomyocyte apoptosis after myocardial IRI (Liu et al.^48^).
SOD1	superoxide dismutase (Cu-Zn)	metabolic enzyme	–	+	IRI: shown to suppress IRI in murine cardiac grafts and vulnerable cerebral neurons (Chan et al.^49^; Tanaka et al;^50^).
SORD	sorbitol dehydrogenase	metabolic enzyme	–	+	AKI: enzyme in the polyol pathway. Polyol pathway knockout or inhibition reduced AKI in mouse (Andres-Hernando et al.^51^).

List of RBPs identified as differentially RNA binding in the porcine eRIC datasets, including gene name, protein name, and PANTHER protein class.^36^^,^^37^ The table also indicates whether each RBP was identified in human and/or mouse kidney tissue^19^ and their reported association with AKI. Full table is provided in Table S24.

## Discussion

In this study, we present comprehensive RNA-protein interactomes for pig and human kidney tissues, bridging basic RBP research with clinically relevant insights into kidney disease and marking an important step toward the translation of RBP biology into clinical settings. To facilitate exploration and visualization of the datasets, we composed an interactive online interface accessible at: [Database]: [https://shiny.cecad.uni-koeln.de/porcine_rbpome/].

Using eRIC, we first identified RBPs in pig and human kidneys at baseline. High-confidence RBPs were defined as those significantly enriched in crosslinked samples (Student’s *t* test, positive log2 fold change), detected exclusively in crosslinked samples, or with a human RBP2GO score >20,^31^ indicating a previously known RBP. This stringent filtering strategy minimized false positives, resulting in the identification of 818 kidney RBPs in pigs and 561 in humans.

Additionally, we investigated IRI-induced changes in the RNA interactome using kidneys from a porcine cardiopulmonary resuscitation model relevant to human disease. The goal was to obtain samples with a condition of mild AKI showing clear signs of IRI but avoiding massive damage to gain mechanistic insight rather than observing the downstream consequences of cell death at a point of no return. IRI-induced damage patterns were confirmed by kidney function parameters (Figures 1B and 1C), urinary biomarkers (Figure 1D), transcriptomic and proteomic profiling (Figure 2B; Tables S4, S5, S6, S7, S8, and S9). Consistent with previous studies, we observed changes in metabolic activity, activation of immunological processes and apoptosis in the IRI group. In our model, the average time to return of spontaneous circulation was 17.6 min following cardiac arrest. This is shorter compared to models employing pedicle clamping, where ischemia typically exceeds 25 min.^52^ This, together with histological findings, supports an early-stage AKI phenotype (Figures 1B–1D; Figure S1). Such a phenotype, characterized by the absence of necrosis, provides a valuable framework for dissecting mechanistic aspects of injury beyond cell death. Comparison of marker genes from healthy and injured PT cells,^26^ to the pig kidney transcriptome further confirmed a loss of healthy PT signatures and a gain of injured PT characteristics (Figure 2C). The transcriptional profile of the damaged porcine kidneys showed high concordance with early-stage rodent and human AKI models (Figure 2D).^27^^,^^28^ Therefore, our porcine urinary proteome, kidney proteome, and kidney transcriptome constitute valuable resources for validating pathophysiological changes relevant to human AKI.

The recent adaptation of the eRIC protocol for use in animal tissues such as the kidney has substantially expanded the number of organ-specific RBPs and underscored the widespread RNA-binding capacity of metabolic enzymes.^19^ Until now, kidney tissue RBPs had been characterized only in mice.^19^ The RNA interactome data generated from pig and human kidneys complement and extend our understanding of the renal RBP landscape, revealing a high degree of cross-species conservation among mouse, pig, and human. This evolutionary conservation underscores the functional importance of RNA-protein interactions in maintaining kidney homeostasis and supports the translational relevance of the porcine model for studying human renal disease mechanisms.

In line with findings from other RNA interactome datasets, the PANTHER protein class analysis^36^^,^^37^^,^^38^ of our pig and human kidney RBPomes revealed that RNA processing and translational proteins were the most abundant functional categories. Metabolic enzymes represented the second most strongly enriched protein class (Figure 3C), consistent with accumulating evidence that metabolic enzymes frequently associate with RNA.^19^ Notably, the proportion of metabolic enzymes was even higher among the newly identified RBPs, suggesting potential tissue- and context-specific regulation: 42% of novel pig and 41% of the novel human kidney RBPs were metabolic enzymes (Table S21). Physical interaction of the metabolic enzymes FH, EHHADH, BHMT, and SORD with RNA was confirmed using a biotin-based visualization of RBP-coupled RNA (Figure S8).^42^

The RNA-binding capacity of metabolic enzymes is well established and has become an increasing focus of recent studies. Prominent examples include adenosine kinase, argininosuccinate synthase, cytosolic aconitase, delta-1-pyrroline-5-carboxylate synthase, enolase 1, and thymidylate synthase, among others.^16^^,^^34^^,^^35^^,^^53^^,^^54^ In line with this, our datasets also identified several transporter proteins as novel RBPs, including aquaporin-1 (AQP1) and the large neutral amino acids transporter small subunit 2 (SLC7A8) in pig kidney, as well as voltage-dependent calcium channel gamma-2 subunit (CACNG2) and sodium-dependent phosphate transport protein 4 (SLC17A3) in human kidney. Although a few transporters such as monocarboxylate transporter 4 (MCT4) and ADP/ATP translocase 2 have previously been reported to bind RNA,^34^^,^^35^^,^^55^ most transport proteins remain to be experimentally validated as RBPs. Given the critical role of transporters in maintaining renal homeostasis and mediating reabsorption processes,^56^ their association with RNA may represent a previously unrecognized regulatory mechanism. These findings open new avenues for functional studies exploring RNA-dependent modulation of transporter activity in the kidney.

We identified 47 RBPs associated with kidney pathophysiology (Figure 3D; Table S22), representing promising candidates for future investigation in specific disease contexts. Notably, these RBPs encompass diverse functional categories beyond canonical RNA processing, including metabolic enzymes and transporters. Several of the identified RBPs have previously been linked to the pathophysiology of AKI,^10^^,^^57^^,^^58^^,^^59^ among them the canonical RBP ELAVL1, which is known to be upregulated following IRI.

Understanding the modulation of RBPs during IRI is crucial for the identification of disease-driving mechanisms and exploring their potential as therapeutic targets in human AKI. As previously demonstrated, cellular stressors such as hypoxia can alter the RNA-binding behavior of RBPs.^12^^,^^16^^,^^18^ Here, we identified 27 RBPs with significantly altered RNA-binding following IRI in the porcine kidney (Figure 4D; Table S24). These differentially bound RBPs participate in essential cellular processes, including metabolic enzymatic activity, RNA metabolism, translation, and transcriptional regulation. Notably, several of these RBPs have already been implicated in AKI or ischemic injury. For example, the peroxisomal enzyme EHHADH, which catalyzes steps in the β-oxidation of very long-chain fatty acids,^57^ exhibited increased RNA association following IRI. Loss of EHHADH has previously been associated with male-specific PT injury in mice^46^ and with accelerated tubulointerstitial injury in male diabetic kidney disease.^47^ Given this sex-specific phenotype, it remains uncertain whether our observations can be generalized to other sexes, and such applicability appears unlikely. Consistent with our findings in pigs and humans, this metabolic enzyme, representing a non-canonical RBP, was previously identified as an RBP in mouse kidney tissue.^19^ Additionally, we corroborated EHHADH's capability to bind RNA using the biotin-based visualization of RBP-coupled RNA (Figure S8).

Another example is BHMT, a metabolic enzyme involved in the conversion of homocysteine to methionine within sulfur amino acid metabolism,^57^ which also showed increased RNA binding in response to IRI. Restriction of sulfur-containing amino acids in rodents has been shown to confer protection against IRI,^44^^,^^60^ suggesting that BHMT and cysteine catabolism pathways may serve as promising targets for AKI protection. As a non-canonical RBP, we also validated BHMT’s capability to bind RNA using the biotin-based visualization of RBP-coupled RNA (Figure S8). Interestingly, both EHHADH and BHMT exhibit particularly high expression in the proximal tubule, highlighting this nephron segment as a focal site for RNA-mediated regulation of metabolic stress responses.

It has been demonstrated that the metabolic activity of an RBP can be modulated by its interacting RNA.^16^ Accordingly, the altered RNA binding observed for these RBPs may directly influence their enzymatic functions, thereby contributing to metabolic dysregulation and exacerbation of kidney injury. However, the precise molecular mechanisms underlying these effects remain to be elucidated for each individual protein.

Notably, several RBPs with increased RNA binding after IRI are components of the translational machinery, including multiple eIF3 subunits and ribosomal proteins (Figure 4D; Table S24). Although this observation may initially appear inconsistent with the global translational repression typically associated with cellular stress, increased RNA binding of these factors should not be interpreted as evidence of enhanced protein synthesis, as eRIC measures RNA-binding activity rather than translational output. Instead, increasing evidence indicates that many translation-associated RBPs also mediate selective translational control of stress-responsive mRNAs. For example, eIF3 subunits can promote either translational activation or repression in a transcript- and context-dependent manner, and eIF3d has been implicated in metabolic stress-induced translational switching.^61^^,^^62^ Together with the enrichment of biological processes related to negative regulation of translation and cellular stress responses in our transcriptomic dataset (Table S7), our findings are consistent with stress-induced translational reprogramming rather than enhanced global protein synthesis. Direct assessment of translational activity using approaches such as polysome profiling or ribosome profiling will be an important objective for future studies.

This work provides a significant advance in our understanding of RBPs in kidney tissue and suggests that non-canonical RBPs extend beyond metabolic enzymes to include novel, and in part kidney-specific, protein classes such as transporters. The functional implications of this expanded RBP repertoire represent an exciting direction for future studies. Moreover, in-depth analysis of the RBPs modulated in our porcine IRI model, together with the identification of their RNA targets, will yield valuable insights into their pathophysiological role in AKI. Such knowledge could inform the development of new therapeutic strategies aimed at modulating RNA-protein interactions. Potential approaches may include targeting the RNA partners of specific RBPs or, in the case of enzymatic RBPs, harnessing RNA molecules to regulate enzymatic activity—a concept referred to as riboregulation.^11^^,^^16^^,^^63^

RBPs are increasingly recognized as potentially druggable regulators of gene expression, and multiple approaches to modulate RNA-protein interactions, including antisense oligonucleotides, small molecules, aptamers, and synthetic peptides, are under active investigation.^64^^,^^65^ Although the present study does not establish causal roles for the RBPs identified in renal IRI, the comprehensive RBPome resource generated here provides a foundation for prioritizing candidates for future mechanistic studies. Determining whether modulation of specific RNA-protein interactions can influence the development or progression of AKI will require functional validation in appropriate experimental models.

### Limitations of the study

This study has several limitations. First, the sample size was limited due to the use of a large animal model. Although sufficient to identify robust changes, larger cohorts would increase statistical power and improve the generalizability of the findings. Second, sex was not evaluated as a biological variable, as all human and porcine samples analyzed were derived from male individuals. Although sex-specific differences cannot be excluded, we anticipate that the major molecular mechanisms identified here are not sex specific. Third, steady-state RNA- seq cannot distinguish whether the transcriptomic remodeling observed after IRI reflects altered transcription, changes in mRNA stability, or both. Consequently, although we demonstrate widespread remodeling of the renal RBPome, our data do not establish a causal relationship between altered RNA binding and transcriptome remodeling. Finally, eRIC selectively captures RBPs associated with polyadenylated RNAs and therefore does not detect interactions with non-polyadenylated transcripts. Comprehensive characterization of the entire RBPome will require unbiased tissue-compatible approaches, which were not available when this study was initiated.

## Resource availability

### Lead contact

Requests for further information and resources should be directed to and will be fulfilled by the lead contact, Roman-Ulrich Müller (roman-ulrich.mueller@med.uni-duesseldorf.de).

### Materials availability

This study did not generate new unique reagents.

### Data and code availability

• An interactive online database, integrating the proteome, transcriptome, and eRIC data of both the pig and human kidneys, was developed using the “shiny” package in R. The database is accessible at the following link: https://shiny.cecad.uni-koeln.de/porcine_rbpome/.
• RNA-seq data generated in this study have been deposited in the ArrayExpress collection of BioStudies under accession number E-MTAB-14235 (https://www.ebi.ac.uk/biostudies/arrayexpress/studies/E-MTAB-14235). The dataset is publicly available.
• Proteomics data generated in this study have been deposited in the ProteomeXchange Consortium via the PRIDE repository (https://www.ebi.ac.uk/pride/) under the following accession numbers: PXD058647 for porcine kidney eRIC (3270) and proteome (3179) datasets (https://www.ebi.ac.uk/pride/archive/projects/PXD058647); PXD058685 for human kidney eRIC (3309) and proteome (3353) datasets (https://www.ebi.ac.uk/pride/archive/projects/PXD058685); and PXD058643 for porcine urine proteomics data (https://www.ebi.ac.uk/pride/archive/projects/PXD058643). All datasets are publicly available.
• Microscopy data reported in this paper will be shared by the lead contact upon request
• This paper does not report original code.
• Any additional information required to reanalyze the data reported in this paper is available from the lead contact upon request.

## Acknowledgments

This work was supported by the 10.13039/501100001659Deutsche Forschungsgemeinschaft (DFG, 10.13039/501100001659German Research Foundation IG 124/2-1 to M.I. and under Germany's Excellence Strategy - EXC 2030 - 390661388). R.-U.M. received further support from the 10.13039/501100001659DFG (MU 3629/10-1/, INST 216/1314-1/SFB 1678, INST 216/1163-1 FUGG, DFG MU 3629/6–1, and MU 3629/3-2/CRU 329) and the German Ministry of Research (10.13039/501100002347BMBF RNA-STAB). P.B. is supported by the 10.13039/501100001659DFG (project IDs 322900939 & 445703531), European Research Council (ERC Consolidator Grant no 101001791), the 10.13039/501100002347BMBF (STOP-FSGS-01GM2202C), and the Innovation Fund of the 10.13039/501100014840Federal Joint Committee (Transplant.KI, no. 01VSF21048). The German Federal Ministry for Economic Affairs and Energy funded S.-R.F., B.P., T.N., and P.R. based on a decision of the German Parliament (grant no. ZF4049311TS9). We thank the CECAD Proteomics Facility for the analysis of mass spectrometric data. This work was supported by the large instrument grant INST 216/1163-1 FUGG by the 10.13039/501100001659German Research Foundation (10.13039/501100001659DFG Großgeräteantrag). We thank Serena Greco-Torres and the team from the Experimental Medicine Department for excellent technical assistance. We would also like to thank the Department of Urology, Uro-Oncology, Robot-Assisted and Specialized Urologic Surgery, University Hospital of Cologne for support in obtaining human kidney tissue and Heike Löser (Institute of Pathology, University Hospital of Cologne) for support regarding the BioMaSOTA biobank.

## Author contributions

Conceptualization was carried out by M.I. and R.-U.M. Tissue samples were collected by S. Hinrichs, M.I., C.R., T.N., B.P., and P.R.. Experiments were performed by S. Hinrichs, S. Haselhorst, B.W., S.-R.F., T.N., B.P., C.R., P.R., and M.I. Data acquisition was undertaken by M.I., S. Hinrichs, S.M., and F.L. Data analysis was conducted by S. Hinrichs, K.B., F.L., H.G., S.M., and P.B. Reagents and resources were provided by T.B., B.S., M.I., and R.-U.M. Original draft of the manuscript was written by S. Hinrichs and M.I. All authors contributed to manuscript review and editing.

## Declaration of interests

The authors declare no competing interests.

## STAR★Methods

### Key resources table

**Table:** 

REAGENT or RESOURCE	SOURCE	IDENTIFIER
**Antibodies**		

Monoclonal ANTI-FLAG M2 Antibody	Sigma-Aldrich	Cat. no. F1804; RRID:AB_262044
Normal mouse IgG	Santa Cruz	Cat. no. sc-2025; RRID:AB_737182

**Chemicals, peptides, and recombinant proteins**		

Carboxylated M-PVA C11 Magnetic Beads 50 mg/mL	Perkin Elmer	Cat. no. CMG-203
LNA2.T capture probe:/5AmMC6/+TT + TT + TT + TT + TT + TT + TT + TT + TT + TT (+T: LNA thymidine, T: DNA thymidine)	Exiqon-Qiagen	N/A
RNase 1 (10 U/μL)	Thermo Scientific	Cat. no. EN0601
Benzonase Nuclease	EMD Millipore	Cat. no. 70746–3
Ambion RNase I (100 U/μL)	Invitrogen	Cat. no. AM2294
Dynabead Protein G	Invitrogen	Cat. no. 10003D
pCp-Biotin	Jena Bioscience	Cat. no. NU-1706-BIO
T4 RNA Ligase	BioLabs	Cat. no. M0437M

**Critical commercial assays**		

Direct-zol RNA Miniprep Kit	Zymo research	Cat. no. R2052
SilverQuest Staining Kit	Invitrogen	Cat. no. 45–1001
Chemiluminescent Nucleic Acid Detection Module Kit	Thermo Scientific	Cat. no. 89880

**Deposited data**		

Shiny app “porcine rbpome”	[Database]: [https://shiny.cecad.uni-koeln.de/porcine_rbpome/]	N/A
Raw RNA-seq data	BioStudies	E-MTAB-14235 https://www.ebi.ac.uk/biostudies/arrayexpress/studies/E-MTAB-14235
Raw proteomic data	PRIDE	PXD058647 for porcine kidney eRIC and proteome https://www.ebi.ac.uk/pride/archive/projects/PXD058647 PXD058685 for human kidney eRIC and proteome https://www.ebi.ac.uk/pride/archive/projects/PXD058685 PXD058643 for porcine urine https://www.ebi.ac.uk/pride/archive/projects/PXD058643

**Experimental models: Cell lines**		

HEK293T	ATCC	CRL-3216

**Oligonucleotides**		

Primer for plasmid: FH (forward primer) CTCCGGCGGCGGCGAATTCACGCGT taccgagcacttcggctcc	Integrated DNA Technologies	N/A
Primer for plasmid: FH (reverse primer) CGGGCCCTCTAGACTCGAGCGGCCGC ctttggacccagcatgtcct	Integrated DNA Technologies	N/A
Primer for plasmid: EHHADH (forward primer) cgaACGCGTGCTTTCGGAGGGGGACTAGAG	Integrated DNA Technologies	N/A
Primer for plasmid: EHHADH (reverse primer) cgaGCGGCCGCCAATTTACTGCTAGGGGAGCCTG	Integrated DNA Technologies	N/A
Primer for plasmid: BHMT (forward primer) cgaACGCGTCCACCCGTTGGGGGCAAA	Integrated DNA Technologies	N/A
Primer for plasmid: BHMT (reverse primer) cgaGCGGCCGCCTGTGATTTGAATTTTTGTTTTTC AAAGAGCTCT	Integrated DNA Technologies	N/A
Primer for plasmid: SORD (forward primer) ACGCGTGCGGCGGCGGCCAAGCCC	Integrated DNA Technologies	N/A
Primer for plasmid: SORD (reverse primer) GCGGCCGCTCAGGGATTCTGGTCACTGGGGTCA CACTTGAGCATGAT	Integrated DNA Technologies	N/A

**Recombinant DNA**		

Plasmid DNA Inserts	N/A	See Table S26

**Software and algorithms**		

RStudio	https://posit.co/downloads/	N/A
R (v4.x)	https://www.r-project.org/	N/A
Graphpad PRISM 10	https://www.graphpad.com/features	N/A
Perseus 1.6.15	https://maxquant.net/perseus/	N/A

### Experimental model and study participant details

All animal procedures were conducted in compliance with European (European Union directive 86/609/EEC), national and institutional guidelines, and were approved by the local governmental authorities (LANUV 81–02.04.2019.A072). In line with the 3 R principle, we used kidneys of pigs that were originally part of a cardiopulmonary resuscitation study, conducted by the Anesthesiology Department at University Hospital Cologne in collaboration with the Experimental Medicine Department. This study utilized kidney tissue from six male German Large White and Piétrain F1 hybrids, aged 3–4 months and weighing approximately 37 kg. One kidney was harvested from each of four pigs that underwent cardiopulmonary resuscitation following induced cardiac arrest (IRI group), while both kidneys were collected from two control animals (control group). Urine and serum samples were also obtained from these animals before euthanasia, along with samples from three additional pigs prior to cardiac arrest.

The human tissue was acquired as part of the standardized biobank of the University Hospital Cologne (BioMaSOTA), and its use for molecular research was approved by the local ethics committee of the University of Cologne (#20–1206). Written informed consent was received prior to participation. All experiments conform to the relevant regulatory standards. Healthy kidney tissue was obtained from three male patients who underwent tumor nephrectomy. The tissue was macroscopically and microscopically tumor-free, with less than 15% affected by interstitial fibrosis and tubular atrophy. All patients provided informed consent, and an experienced nephropathologist assisted in the tissue collection. The mean age of the patients was 49 years, with a mean creatinine/eGFR of 93.3 mL/min.

Sex was not analyzed as a biological variable in this study. All tissue samples used in this study from Large White × Piétrain F1 hybrids and humans were obtained from male individuals. The pig tissue used in this study originated from a separate study, and therefore their sex could not be controlled. The three male tumor nephrectomy patients had the best renal function among the small group consenting to tissue sampling. We expect the findings to be applicable beyond a single sex unless otherwise stated.

### Method details

#### Porcine cardiopulmonary resuscitation model

Male German Large White and Piétrain F1 hybrids, aged 3–4 months and weighing approximately 37 kg were anesthetized and prepared for surgery with intubation, urine drainage, cardiovascular monitoring and equipment for fluid and drug therapy. Cardiac arrest was induced by alternating current, placed directly to the heart. After 10 min without intervention, resuscitation was performed with an automated CPR device (corpuls cpr; corpuls). The workflow for resuscitation was based on the ERC (European Resuscitation Council) guidelines.^66^ Animals returning to spontaneous circulation were stabilized and observed for 5 h. In the control group, no cardiac arrest was induced and the pigs were observed for an equivalent amount of time (Figure 1A). Blood was aspirated from a central venous catheter in an S-Monovette Lithium heparin gel (Sarstedt; 01.1634). The urine was taken from the urine drainage system. Both were centrifuged and the supernatant was stored at −20 °C until further use. After the observation period, both groups were euthanized. The kidneys were removed and perfused with 400 mL Jonosteril Infusion solution (Fresenius) and 10.000 units Heparin-Sodium (Braun; 2147217). Kidney tissue samples were snap frozen in liquid N2 and stored at −80 °C until further use. Sections of the kidneys were fixed in 4% formaldehyde for histological analysis.

#### Histological assessment of tubular damage

Formalin fixed and paraffin-embedded kidney sections were cut to 1 μm thick slices. After PAS staining, the kidney sections were assessed in a blinded manner for pathological findings by two experienced nephropathologist (Prof. Boor; Dr. Göbel). Specific focus was given to signs of tubular injury consistent with ischemia-reperfusion injury, including tubular dilation, vacuolization, epithelial flattening and loss of brush border (Figure S1).^67^^,^^68^

#### Serum and urine analyses

Creatinine measurements of serum (Creatinine Plus Test Ver. 2 (Roche Diagnostics, product number) as well as serum urea measurements (Urea test (Roche Diagnostics, product number) were conducted by the central laboratory of the University Hospital of Cologne using a Cobas C 702 (Roche Diagnostics, Mannheim, Penzberg, Germany). The IRI group comprised four samples (one per animal), and the control group comprised five samples (one per animal), three of which were collected prior to induction of cardiac arrest as additional controls. Statistical significance was calculated with an unpaired *t* test (GraphPad Prism 9.3.1, GraphPad Software, Boston, Massachusetts USA, www.graphpad.com) (Figures 1B and 1C). A *p*-value of <0.05 was considered significant.

#### Urine proteomics

Urine proteins were digested according to the SP3 protocol.^69^ The IRI group comprised four samples (one per animal), and the control group comprised five samples (one per animal), three of which were collected prior to induction of cardiac arrest as additional controls. Samples were analyzed by the CECAD Proteomics Facility on an Orbitrap Exploris 480 (Thermo Scientific, granted by the German Research Foundation under INST 1856/71–1 FUGG) mass spectrometer equipped with a FAIMSpro differential ion mobility device that was coupled to an Evosep One HPLC (Evosep). Samples were loaded onto Evotips according to the manufacturers standard operating procedure and analyzed using the 30 samples per day method with the EV1140 Performance Column (Evosep) and a stainless-steel emitter. The FAIMS pro was operated at −50 V compensation voltage and electrode temperatures of 99.5 °C for the inner and 85 °C for the outer electrode. MS1 scans were acquired from 399 m/z to 1001 m/z at 15 k resolution. Maximum injection time was set to 22 ms and the AGC target to 100%. MS2 scans ranged from 400 m/z to 1000 m/z and were acquired at 15 k resolution with a maximum injection time of 22 ms and an AGC target of 100%. DIA scans covering the precursor range from 400–1000 m/z and were acquired in 50 × 12 m/z staggered windows. All scans were stored as centroid. Thermo raw files were demultiplexed and transformed to mzML files using the msconvert module in Proteowizard. All samples were analyzed in DIA-NN 1.8.1.^70^ The UniProt pig proteome database (UP000008227, downloaded 18/03/2022) was used for library building with settings matching acquisition parameters and the match-between-runs function enabled. Here, a larger porcine urine dataset was used to refine the library for a second search of the sample data. DIA-NN was run with the additional command line prompts “—report-lib-info” and “—relaxed-prot-inf”. Further output settings were: filtered at 0.01 FDR, N-terminal methionine excision enabled, maximum number of missed cleavages set to 1, min peptide length set to 7, max peptide length set to 30, min precursor m/z set to 400, max precursor m/z set to 1000, cysteine carbamidomethylation enabled as a fixed modification. Afterward, DIA-NN output was further filtered on library q-value and global q-value ≤ 0.01 and at least two unique peptides per protein using R (4.1.3). Missing values were replaced by ImputeLCMD package in R using the method impute.MinDet with default settings.^71^ Intensities were then uploaded to Perseus 1.6.15.0.^72^ The rows were filtered, to contain at least three valid values (measured, not imputed) in at least one of the experimental groups. Student's *t* test was performed with an S_0_ of 0.2 by default Perseus settings. A q-value of <0.05 was considered significant. The dataset is provided in Table S1.

#### Kidney tissue lysis for eRIC

Frozen pig or human kidney was cryosectioned into 30 μm thick slices. Every second slice was UV (254 nm) crosslinked with 1 J/cm^2^. The crosslinked and non-crosslinked sections were homogenized in 17 mL ice-cold eRIC lysis buffer (20 mM Tris-HCl (pH:7.5), 500 mM LiCl, 0.5% (w/v) LiDS, 1 mM EDTA, 5 mM DTT, 1× cOmplete EDTA-free Protease Inhibitor Cocktail (Merck), in ultra-pure RNase free water), snap frozen in liquid N2 and stored at −80 °C until further use.^19^ For the porcine eRIC, two times 0.65 mg kidney tissue were lysed in 17 mL eRIC lysis buffer (crosslinked and not crosslinked separately), for the human eRIC 0.65 mg was used.

#### Kidney tissue samples for whole proteome analysis

20 μL non crosslinked kidney lysate were incubated with 80 μL ice-cold acetone for 15 min at −80°C and overnight at −20°C. The samples were centrifuged for 15 min at 16.000 g and the supernatant was removed. 0.5 mL ice-cold acetone was added to the protein pellet and the sample were centrifuged for 5 min at 16.000 g. This step was repeated. The protein pellet was air dried for 5 min, and resuspended in PBS +5% SDS. The samples were incubated for 10 min at 95°C and sonicated for 10 cycles (30s/30s) (bioruptor plus/diagenode). The protein concentration was measured with the Pierce BCA Protein Assay Kit (Thermo scientific; 23227). 20 μg of protein were reduced by adding DTT to a final concentration of 5 mM and incubating at 55°C for 30 min. Then, the samples were alkylated by adding CAA to 40 mM and incubating for 30 min at RT in the dark. Proteins were digested using SP3 protocol.^69^

#### Analysis of the porcine kidney proteome

The IRI group consisted of four samples from four animals and the control group consisted of four samples, including each kidney from two animals. Samples were analyzed by the CECAD Proteomics Facility on an Orbitrap Exploris 480 (Thermo Scientific, granted by the German Research Foundation under INST 1856/71–1 FUGG) mass spectrometer equipped with a FAIMSpro differential ion mobility device that was coupled to an UltiMate 3000 (Thermo Scientific). Samples were loaded onto a precolumn (Acclaim 5 μm PepMap 300 μm Cartridge) for 2 min at 15 μl flow before reverse-flushed onto an in-house packed analytical column (30 cm length, 75 μm inner diameter, filled with 2.7 μm Poroshell EC120 C18, Agilent). Peptides were chromatographically separated at a constant flow rate of 300 nL/min and the following gradient: initial 6% B (0.1% formic acid in 80% acetonitrile), up to 32% B in 72 min, up to 55% B within 7.0 min and up to 95% solvent B within 2.0 min, followed by column wash with 95% solvent B and reequilibration to initial condition. The mass spectrometer was operated in data-dependent acquisition mode with MS1 scans acquired from 350 m/z to 1400 m/z at 60 k resolution and an AGC target of 300%. MS2 scans were acquired at 30 k resolution with a maximum injection time of 60 ms and an AGC target of 300% in a 1.4 Th window and a fixed first mass of 110 m/z. The FAIMS pro was operated with electrode temperatures of 99.5 °C for the inner and 85 °C for the outer electrode and compensation voltage (CV) stepping using −45 V, −60 V and −75 V.^73^ The selected CV's were applied to the MS1 and the dependent MS2 scans. Precursors were selected by a TopN method with *N* = 12 for −45 V, *N* = 8 for −60 V and *N* = 4 for −75 V.All MS1 scans were stored as profile, all MS2 scans as centroid. Whitin each cycle the Faims stept through 3 compensation voltages, namely −45 V for 12 MS/MS scans, −60 V/8 MSMS scans and −75 V (MS7MS scans). Raw data files were split into individual CV traces in Freestyle (Thermo Scientific). All files were processed with Maxquant (version 2.2)^74^ using default parameters against a uniprot Pig proteome database (UP000008227, downloaded 18/03/2022) with the match-between-runs option enabled between replicates. The three CV traces from each sample were treated as fractions. Follow-up analysis was done with label free quantification (LFQ) intensities in Perseus 1.6.15.^72^ Potential contaminants, proteins only identified by site and reversed were filtered out. Intensities were transformed to log2(x). The rows were filtered, to contain at least three valid values in at least one of the experimental groups. Sample IRI 4 was excluded from further analysis due to a lower protein count compared to the other samples. Missing values were replaced from normal distribution by standard Perseus DDA imputation (width 0.3; down shift 1.8) separately for each column. Principal component analysis and Student's *t* test with an S_0_ of 0.2 was performed with default Perseus settings. A q-value of <0.05 was considered significant. The dataset is provided in Table S2. The amount of total and significant identified proteins was based on unique gene names.

#### PANTHER and Gene ontology analysis of porcine proteome

GO biological process, PANTHER Pathway and Reactome analysis was done with PANTHER overrepresentation test (Released 20240226) using Fisher’s exact test with false discovery rate correction and PANTHER version 18.0 Released 2023–08-01, Reactome version 85 Released 2023–05-25 or GO Ontology database https://doi.org/10.5281/zenodo.10536401 Released 2024–01-17 annotation.^33^^,^^34^^,^^36^^,^^37^^,^^38^ An FDR <0.05 was considered significant. The analyzed list contained all significant protein's gene names. All protein’s gene names were used as reference list. The results from the overrepresentation tests are provided in Tables S4, S5, and S6.

#### Analysis of the human kidney proteome

The dataset is based on three samples from three individuals. Samples were analyzed by the CECAD Proteomics Facility on an Orbitrap Exploris 480 (Thermo Scientific, granted by the German Research Foundation under INST 1856/71–1 FUGG) mass spectrometer equipped with a FAIMSpro differential ion mobility device that was coupled to an UltiMate 3000 (Thermo Scientific). Samples were loaded onto a precolumn (Acclaim 5 μm PepMap 300 μm Cartridge) for 2 min at 15 μl flow before reverse-flushed onto an in-house packed analytical column (30 cm length, 75 μm inner diameter, filled with 2.7 μm Poroshell EC120 C18, Agilent). Peptides were chromatographically separated at a constant flow rate of 300 nL/min and the following gradient: initial 6% B (0.1% formic acid in 80% acetonitrile), up to 32% B in 72 min, up to 55% B within 7.0 min and up to 95% solvent B within 2.0 min, followed by column wash with 95% solvent B and re-equilibration to initial condition. The FAIMS pro was operated at −50 V compensation voltage and electrode temperatures of 99.5 °C for the inner and 85 °C for the outer electrode. MS1 scans were acquired from 399 m/z to 1001 m/z at 15 k resolution. Maximum injection time was set to 22 ms and the AGC target to 100%. MS2 scans ranged from 400 m/z to 1000 m/z and were acquired at 15 k resolution with a maximum injection time of 22 ms and an AGC target of 100%. DIA scans covering the precursor range from 400–1000 m/z and were acquired in 60 × 10 m/z windows with an overlap of 1 m/z. All scans were stored as centroid. Samples were analyzed in DIA-NN 1.8.1.^70^ A Swissprot human canonical database (UP000005640, downloaded 18/03/2022) was used for library building with settings matching acquisition parameters and the match-between-runs function enabled. Here, samples are directly used to refine the library for a second search of the sample data. DIA-NN was run with the additional command line prompts “—report-lib-info” and “—relaxed-prot-inf”. Further output settings were: filtered at 0.01 FDR, N-terminal methionine excision enabled, maximum number of missed cleavages set to 1, min peptide length set to 7, max peptide length set to 30, min precursor m/z set to 400, max precursor m/z set to 1000, cysteine carbamidomethylation enabled as a fixed modification. Afterward, DIA-NN output was further filtered on library q-value and global q-value ≤ 0.01 and at least two unique peptides per protein using R (4.1.3). Afterward, analysis of results was performed in Perseus 1.6.15.^72^ N1U intensities were transformed to log2(x) and the rows were filtered, to contain at least two valid values out of three samples. The dataset is provided in Table S18.

#### Porcine kidney transcriptome

The IRI group consisted of four samples from four animals and the control group consisted of four samples, including each kidney from two animals. Frozen kidney was homogenized in TRI Reagent (Signma-Aldrich) and the RNA was isolated using the Direct-zol RNA Miniprep Kit (Zymo research; R2052) with DNase I digestion according to manufacturer’s protocol. Libraries were prepared using the Illumina Stranded TruSeq RNA sample preparation Kit. Library preparation started with 500 ng total RNA. Poly-A selected mRNA was purified and fragmented. The RNA fragments underwent reverse transcription using random primers. This was followed by second strand cDNA synthesis with DNA Polymerase I and RNase H. After end repair and A-tailing, indexing adapters were ligated. The products were then purified and amplified (15 PCR cycles) to create the final cDNA libraries. After library validation and quantification (Agilent Tape Station), equimolar amounts of library were pooled. The pool was quantified by using the Peqlab KAPA Library Quantification Kit and the Applied Biosystems 7900HT Sequence Detection System. The pool was sequenced on the Illumina NovaSeq6000 platform with a PE100 protocol aiming for 30 million clusters per sample. The reads were trimmed for Illumina adapters with Trimmomatic (version 0.36)^75^ using default parameters. Additionally, the first ten bases were cropped because of bad quality. The trimmed reads were mapped to the pig reference genome Sscrofa11.1 with STAR version 2.6.0^76^ using default parameters. The analysis of differential gene expression was performed using the R package DESeq2 (version 1.26.0).^77^^,^^78^ A adjusted *p*-value of <0.05 was considered significant. The total identified 15 722 transcripts were based on a DESeq2 normalized read count (mean across all samples) > 10. The dataset is provided in Table S3.

#### PANTHER and Gene ontology analysis of porcine transcriptome

PANTHER Pathway and reactome analysis was done with PANTHER Overrepresentation Test (Released 20230705) and GO biological process with PANTHER Overrepresentation Test (Released 20240226) using Fisher’s Exact test with false discovery rate correction and GO Ontology database https://doi.org/10.5281/zenodo.10536401 Released 2024–01-17, PANTHER version 18.0 Released 2023–08-01 or Reactome version 85 Released 2023–05-25 annotation.^33^^,^^34^^,^^36^^,^^37^^,^^38^ An FDR <0.05 was considered significant. The analyzed list contained all significant gene names with all gene names as reference list. The results from the Overrepresentation Tests are provided in Tables S7, S8, and S9.

Significantly regulated pig kidney transcripts (IRI vs. control) were compared to previously published regulated transcripts in mouse and human IRI dataset.^27^^,^^28^ Gene names from both datasets were converted to pig gene names by Ensembl BioMart.^79^ The shared transcripts of pig, mice and human IRI data were annotated with PANTHER Pathway.^38^ The list is provided in Table S13.

#### Enhanced RNA interactome capture (eRIC)

The kidney lysate was thawed at 37°C and then incubated at 60°C for 15 min. Samples were placed on ice and fresh DTT was added to 5 mM. The samples were centrifuged with 3 220 rcf at 4°C for 10 min and the supernatant was transferred to a 15 mL DNA LoBind Tube (Eppendorf; 0030122208). Coupled LNA oligo beads (Carboxylated M-PVA C11 Magnetic Beads 50 mg/mL with LNA2.T capture probe:/5AmMC6/+TT + TT + TT + TT + TT + TT + TT + TT + TT + TT (+T: LNA thymidine, T: DNA thymidine))^13^^,^^14^ were washed three times in eRIC lysis buffer and added to the lysate. After 1 h of incubation rotating at 40°C, the beads were captured on a magnet. The supernatant was transferred to a new 15 mL DNA LoBind Tube on ice for a second round of capture. The beads were washed once with eRIC lysis buffer and twice with eRIC washing buffer 1 (20 mM Tris-HCl (pH:7.5), 500 mM LiCl, 0.1% (w/v) LiDS, 1 mM EDTA, 5 mM DTT, in ultra-pure RNase free water), 2 (20 mM Tris-HCl (pH:7.5), 500 mM LiCl, 0.02% (v/v) NP40, 1 mM EDTA, 5 mM DTT, in ultra-pure RNase free water) and 3 (20 mM Tris-HCl (pH:7.5), 200 mM LiCl, 0.02% (v/v) NP40, 1 mM EDTA, 5 mM DTT, in ultra-pure RNase free water). Each washing step was performed with 5 mL prewarmed buffer, rotating at 40°C for 5 min. The beads were resuspended in 220 μL ultra-pure RNase free water and incubated for 5 min at 40°C and 800 rpm in a thermomixer. After capturing the beads on a magnet, the supernatant was removed and the beads were resuspended in 220 μL ultra-pure RNase free water. 20 μL of the sample was removed for “heat-elution”, the rest was resuspended in 150 μL of RNase buffer (10 mM Tris-HCl (pH:7.5), 1 mM MgCl_2_, 0.01% NP-40, 5 mM DTT, 6.5U RNase 1 (Thermo Scientific; EN0601) and 76U Benzonase Nuclease (EMD Millipore; 70746–3) in ultra-pure RNase free water) and incubated for 3 h at 37°C and 800rpm in a thermomixer. The “RNase eluate” was saved in a 1.5 mL microcentrifuge tube and stored on ice. The beads were resuspended in ultra-pure RNase free water. For “heat-elution”, the beads were resuspended in 15 μL ultra-pure RNase free water and incubated for 10 min at 90°C and 800rpm in a thermomixer. The samples were centrifuged and the beads were captured on a magnet. The “heat eluate” was snap frozen and stored at −80 °C until further use.

The capture was repeated on more time with the saved lysate and the two RNase elutions from both captures were pooled. The eluates were cleaned and concentrated with acetone precipitation. 260 μL of RNase eluate was incubated with 1.1 mL ice-cold acetone for 15 min at −80°C and overnight at −20°C. The samples were washed with acetone, resuspended in 5% SDS with 1xPBS, incubated at 95°C and sonicated like described earlier. DTT was added to a final concentration of 5 mM following an incubation at 55°C for 30 min. Then, the samples were alkylated by adding CAA to 40 mM and incubating for 30 min at RT in the dark. Proteins were digested using SP3 protocol.^69^

#### Silver gel analysis

For silver gel analysis of the eRIC samples, lysates were mixed with LDS sample buffer (Nupage, Invitrogen NP0007) and DTT (0.1 M). The samples were incubated for 2 min at 95°C, then loaded and separated on 4–12% Bis-Tris Gels (NuPAGE, Invitrogen; NP0321). The silver staining was performed with the SilverQuest Staining Kit (Invitrogen; 45–1001) according to manufacturer’s protocol (Figure 3A).

#### Analysis of porcine eRIC

The IRI group consisted of four samples from four animals and the control group consisted of four samples, including each kidney from two animals. Samples were analyzed by the CECAD Proteomics Facility on an Orbitrap Exploris 480 (Thermo Scientific, granted by the German Research Foundation under INST 1856/71–1 FUGG) mass spectrometer equipped with a FAIMSpro differential ion mobility device that was coupled to an UltiMate 3000 (Thermo Scientific). Samples were loaded onto a precolumn (Acclaim 5 μm PepMap 300 μm Cartridge) for 2 min at 15 μl flow before reverse-flushed onto an in-house packed analytical column (30 cm length, 75 μm inner diameter, filled with 2.7 μm Poroshell EC120 C18, Agilent). Peptides were chromatographically separated at a constant flow rate of 300 nL/min and the following gradient: initial 6% B (0.1% formic acid in 80% acetonitrile), up to 32% B in 72 min, up to 55% B within 7.0 min and up to 95% solvent B within 2.0 min, followed by column wash with 95% solvent B and reequilibration to initial condition. The mass spectrometer was operated in data-dependent acquisition mode with MS1 scans acquired from 350 m/z to 1400 m/z at 60 k resolution and an AGC target of 300%. MS2 scans were acquired at 30 k resolution with a maximum injection time of 60 ms and an AGC target of 300% in a 1.4 Th window and a fixed first mass of 110 m/z. The FAIMS pro was operated with electrode temperatures of 99.5 °C for the inner and 85 °C for the outer electrode and compensation voltage (CV) stepping using −45 V, −60 V and −75 V.^73^ The selected CV's were applied to the MS1 and the dependent MS2 scans. Precursors were selected by a TopN method with *N* = 12 for −45 V, *N* = 8 for −60 V and *N* = 4 for −75 V.All MS1 scans were stored as profile, all MS2 scans as centroid. Whitin each cycle the Faims stept through 3 compensation voltages, namely −45 V for 12 MS/MS scans, −60 V/8 MSMS scans and −75 V (MS7MS scans). Raw data files were split into individual CV traces in Freestyle (Thermo Scientific). All files were processed with Maxquant (version 2.2)^74^ using default parameters against a uniprot Pig proteome database (UP000008227, downloaded 18/03/2022) with the match-between-runs option enabled between replicates. The three CV traces from each sample were treated as fractions. Follow-up analysis was done with LFQ intensities in Perseus 1.6.15.^72^ Potential contaminants, proteins only identified by site and reversed were filtered out. Intensities were log2 transformed. Two samples (one in IRI, Cl group and one in IRI, nCl group) were excluded from further analysis due to a deviating number of proteins compared to the other samples in their group. The rows were filtered, to contain at least three valid values in at least one of the experimental groups. Missing values were replaced from normal distribution by standard Perseus DDA imputation (width 0.3; down shift 1.8) separately for each column. Homo sapiens homologs were annotated using the first noted protein ID in majority protein ID column with Ensembl BioMart.^79^ Student's *t* test was performed with an S_0_ of 0.2 by default Perseus settings between the experimental groups control crosslinked (Cl) vs. control non-crosslinked (nCl) and IRI Cl vs. IRI nCl. A q-value of <0.05 was considered significant. Proteins were filtered for RBP class 1 and 2 in control and IRI group. A class 1 RBP was defined as a protein with at least 3 valid values in the crossed-linked samples, furthermore a significant student's *t* test with an S0 of 0.2 and a positive fold change (FC) in the crosslinked vs. non-crosslinked samples or not measured in non-crosslinked samples. Class 2 RBPs had at least 3 valid values in the crosslinked samples and a RBP2go human score greater than 20, matched on human homolog gene name. At last, a Student's *t* test was performed with a S_0_ of 0.2 on all RBPs of class 1 and 2 by default Perseus settings between the experimental groups IRI Cl vs. control Cl. The dataset is provided in Table S14. Novel identified RBPs were not listed in RBP2go human or mice, RBPBase all organisms or were annotated as “RNA binding” in GO molecular function.^31^^,^^32^^,^^34^ The amount of total and significant identified proteins was based on unique gene names.

#### Analysis of human eRIC

The dataset is based on three samples from three individuals. Samples were analyzed by the CECAD Proteomics Facility on an Orbitrap Exploris 480 (Thermo Scientific, granted by the German Research Foundation under INST 1856/71–1 FUGG) mass spectrometer equipped with a FAIMSpro differential ion mobility device that was coupled to an UltiMate 3000 (Thermo Scientific). Samples were loaded onto a precolumn (Acclaim 5 μm PepMap 300 μm Cartridge) for 2 min at 15 μl flow before reverse-flushed onto an in-house packed analytical column (30 cm length, 75 μm inner diameter, filled with 2.7 μm Poroshell EC120 C18, Agilent). Peptides were chromatographically separated at a constant flow rate of 300 nL/min and the following gradient: initial 6% B (0.1% formic acid in 80% acetonitrile), up to 32% B in 72 min, up to 55% B within 7.0 min and up to 95% solvent B within 2.0 min, followed by column wash with 95% solvent B and re-equilibration to initial condition. The FAIMS pro was operated at −50 V compensation voltage and electrode temperatures of 99.5 °C for the inner and 85 °C for the outer electrode. MS1 scans were acquired from 399 m/z to 1001 m/z at 15 k resolution. Maximum injection time was set to 22 ms and the AGC target to 100%. MS2 scans ranged from 400 m/z to 1000 m/z and were acquired at 15 k resolution with a maximum injection time of 22 ms and an AGC target of 100%. DIA scans covering the precursor range from 400–1000 m/z and were acquired in 60 × 10 m/z windows with an overlap of 1 m/z. All scans were stored as centroid. Samples were analyzed in DIA-NN 1.8.1.^70^ A Swissprot human canonical database (UP000005640, downloaded 18/03/2022) was used for library building with settings matching acquisition parameters and the match-between-runs function enabled. Here, samples are directly used to refine the library for a second search of the sample data. DIA-NN was run with the additional command line prompts “—report-lib-info” and “—relaxed-prot-inf”. Further output settings were: filtered at 0.01 FDR, N-terminal methionine excision enabled, maximum number of missed cleavages set to 1, min peptide length set to 7, max peptide length set to 30, min precursor m/z set to 400, max precursor m/z set to 1000, cysteine carbamidomethylation enabled as a fixed modification. Afterward, DIA-NN output was further filtered on library q-value and global q-value ≤ 0.01 and at least two unique peptides per protein using R (4.1.3). Afterward, analysis of results was performed in Perseus 1.6.15.^72^ N1U Intensities were log2-transformed and the rows were filtered, to contain at least two valid values out of three crosslinked samples. Missing values were replaced by ImputeLCMD package in R using the method impute.MinDet with default settings.^71^ Principal component analysis was performed by default Perseus settings. Student's *t* test was performed with an S_0_ of 0.2 by default Perseus settings between the crosslinked and non-crosslinked samples. A q-value of <0.05 was considered significant. Again, proteins were filtered for RBP class 1 and 2 as defined for the pig eRIC dataset. The dataset is provided in Table S15. Novel identified RBPs were not listed in RBP2go human or mice, RBPBase all organisms or were annotated as “RNA binding” in GO molecular function.^31^^,^^32^^,^^34^ The amount of total and significant identified proteins was based on unique gene names.

#### Interspecies comparison of RBPs

RBPs from porcine and human kidney eRIC (Tables S14 and S15) were overlapped with mouse kidney eRIC RBPs^19^ (from RBPbase Hs^32^) in a Venn diagram in Figure 3B (Table S19).

#### Molecular cloning

All plasmids used in this study were generated by cloning inserts into a triple FLAG-tagged pcDNA6 backbone using the high-fidelity restriction enzymes MluI (NEB, R3198L) and NotI (NEB, R3189L). Inserts were obtained by PCR amplification from cDNA or pre-existing plasmids (Table S26). For each construct, the insert sequence and the vector regions flanking the cloning sites were verified by Sanger sequencing to confirm sequence integrity, exclude cloning-induced mutations, and ensure that the insert was in the correct reading frame.

#### Cell culture and transfection

Human HEK293T cells (ATCC) were cultured in DMEM (GlutaMAX, Gibco) supplemented with 10% fetal bovine serum (FBS) at 37°C in a humidified atmosphere containing 5% CO_2_. Cells were routinely tested for mycoplasma contamination using a PCR-based detection kit (PromoKine), and all cultures were confirmed to be mycoplasma-free. Transfections with RBP expression plasmids were performed in cells at 60–80% confluence using the calcium phosphate method.^80^

#### Biotin-based visualization of RBP-coupled RNA

HEK293T cells transfected with pcDNA6 expressing triple FLAG-tagged genes of interest were UVC (with 250mJ/cm2) crosslinked, resuspended in lysis buffer (150 mM NaCl; 50 mM Tris-HCl pH7.5; 1% Triton X-, 1× cOmplete EDTA-free Protease Inhibitor Cocktail (Merck)), and homogenized through a 23G needle on ice. Samples were sonicated (Biorupter Pico; 10 intervals: 30 s on/off). Lysates were cleared by centrifugation (16,000 × g; 10 min) and treated with 8 U/mL DNase I (NEB, M0303L) and either 40 U/mL RNase I (Invitrogen, AM2294) or 400 U/mL for 15 min shaking at 800rpm and 37°C. Immunoprecipitation was performed with anti-FLAG M2 (Merck, F1804) or normal mouse IgG (Santa Cruz, sc-2025) coupled to Protein G Dynabeads (Invitrogen, 10004D) over night at 4°C. Beads were washed three times with ice-cold high salt washing buffer (50 mM Tris-HCl pH 7.4, 1 M NaCl, 1% (v/v) Igepal CA-630, 1 mM EDTA, 0.1% (v/v) SDS and 0.5% (w/v) sodium deoxycholate) and three times with wash buffer (20 mM Tris-HCl pH 7.4, 10 mM MgCl2, 0.2% (v/v) Tween 20 and 5 mM NaCl). Beads were resuspended in FastAP Buffer Reaction mixture (3 U FastAP (Thermo Scientific, EF0651), 80 U Murine RNase Inhibitor (NEB, M0314L), 4U Turbo DNase (Invitrogen, AM2238), 1× FastAP Buffer (Thermo Scientific, EF0651)) with an incubation for 10 min at 37°C. Then, T4 PNK Buffer Reaction mixture (1U T4 PNK (NEB, M0201S), 1× T4 PNK Buffer (70 mM Tris-HCl, pH 6.5; 10 mM MgCl2) was added with another incubation for 20 min at 37°C for dephosphorylation of 5′ and 3′ end RNAs. Beads were then washed again twice with ice-cold high salt washing buffer and three times with wash buffer. After that, the biotin-labeling was performed using biotinylated cytidine (bis)phosphate (pCp-Biotin) with T4 RNA Ligase Reaction mixture (18,7 nM pCp-Biotin (Jena Bioscience, NU-1706-BIO), 1 mM ATP (NEB, P0756S), 3.5% (v/v) DMSO, 0.025% (v/v) Tween 20, 1× T4 RNA Ligation Buffer w/o DTT (NEB, B0216S), 20% PEG8000 (NEB, B1004S), 20U Murine RNase Inhibitor (NEB, M0314L) and 30U T4 RNA Ligase 1 (NEB, M0437M)). The labeling was carried out for 2 h at 16°C. Beads were washed again twice with ice-cold high salt washing buffer, three times with wash buffer and after that boiled with NuPAGE LDS Sample Buffer (Invitrogen, NP0007) and 100 mM DTT. The samples were resolved on 4%–12% bis-tris gels (ThermoFisher) and transferred onto Protran Nitrocellulose membrane (Schleicher and Schuell). The biotinylation was visualized with the Chemiluminescent Nucleic Acid Detection Module Kit (Thermo Scientific, Cat. no. 89880). IP efficiency was controlled with anti-FLAG M2 western blotting (Merck, F1804).

#### Schematics and illustrations

Schematics and illustrative figures presented in this study were created using Generic Diagramming Platform (GDP). All figures were exported and published in accordance with Generic Diagramming Platform (GDP)'s academic publication guidelines and licensing agreements.

### Quantification and statistical analysis

Transcriptomic differential gene expression analysis was conducted using the DESeq2 package (v1.26.0) in R.^77^^,^^78^ Principal component analysis and Student's *t*-tests of proteomic datasets were conducted using default Perseus settings, a q-value of <0.05 was considered significant. Statistical details of all experiments can be found in the figure legends if shown and/or in the method details section, including the statistical tests used, exact value of n, what n represents.

Gene Ontology (GO) biological process, PANTHER Pathway and Reactome analyses were conducted using the PANTHER Overrepresentation Test, which included Fisher's exact test with false discovery rate (FDR) correction.^33^^,^^34^^,^^36^^,^^37^^,^^38^ An FDR <0.05 was considered significant. Statistical details of all experiments can be found in the figure legends if shown and/or in the method details section, including the statistical tests used, exact value of n, what n represents. The PANTHER protein class was annotated using the PANTHER classification system, and GO biological processes were assigned based on the GO Ontology database.^33^^,^^34^^,^^35^^,^^37^

The percentage of regulated differentially expressed genes (DEG's) of healthy PT (healthy S1, healthy S2, healthy S3), injured PT (injured S1/2, injured S3) and severe injured PT from a snRNAseq dataset^26^ in up or downregulated porcine transcripts was calculated (Figure 2C). Significant difference between the found DEG's in up- and downregulated transcripts was calculated with Fisher Exact Test in a 2 × 2 contingency table (Table S11). A *p*-value of <0.05 was considered significant. Statistical details of the Fisher Exact Test and porcine transcriptome can be found in the figure legends and/or in the method details section, including the statistical tests used, exact value of n, what n represents. Mouse DEG's from a snRNAseq dataset^26^ were converted to porcine gene names by Ensembl BioMart.^79^

To identify differentially RNA binding RBPs, we compared the expression change due to IRI in the proteome (IRI vs. control) and eRIC dataset (IRI crosslinked vs. control crosslinked). An RBP was identified across the two datasets by its first noted protein ID in “Majority protein ID” column. RBPs needed to be significantly regulated with an FC > 1/<-1 in one of the datasets and not regulated with a *p*-value > 0.1 and an FC between 0.4 and −0.4 in the other dataset (Figures 4A and 4B). Statistical details of the porcine proteome and eRIC dataset can be found in the method details section, including the statistical tests used, exact value of n, what n represents.

Publicly available human kidney single-nucleus RNA sequencing (snRNA-seq) data were used to assess the cell-type-specific expression of differentially RNA-bound RBPs, with a focus on proximal tubule cells.^43^ For each gene, its expression rank within proximal tubules (relative to all transcripts in that cell type) was compared to its expression rank in non-tubular cell types (endothelial cells, fibroblasts, leukocytes, glomerular cell types, parietal epithelial cells) and other tubular segments (connecting tubule, distal tubule, collecting duct, thick ascending limb). Proximal tubule specificity was calculated as: Log_2_(1/(ranking in proximal tubules/mean ranking of non-tubules or tubules)). This metric reflects the degree to which a gene is selectively expressed in proximal tubule cells relative to other renal cell types (Figure 4E).

### Additional resources

An interactive online database, integrating the proteome, transcriptome, and eRIC data both the pig and human kidneys, was developed using the “shiny” package in R. The database is accessible at the following link: [Database]: [https://shiny.cecad.uni-koeln.de/porcine_rbpome/].

## Supplemental information

Document S1. Figures S1–S9

Table S1. Mass spectrometry analysis of porcine urine proteome, related to Figure 1

Table S2. Mass spectrometry analysis of the porcine kidney proteome, related to Figure 2

Table S3. RNA bulk sequencing analysis of porcine kidney transcriptome, related to Figure 2

Table S4. PANTHER overrepresentation test of GO biological processes for regulated porcine kidney proteins compared to the entire proteome, related to Figure 2

Table S5. PANTHER overrepresentation test of PANTHER pathways for regulated porcine kidney proteins compared to the entire proteome, related to Figure 2

Table S6. PANTHER overrepresentation test of PANTHER Reactome pathways for regulated porcine kidney proteins compared to the entire proteome, related to Figure 2

Table S7. PANTHER overrepresentation test of GO biological processes for regulated porcine kidney transcripts compared to whole transcriptome, related to Figure 2

Table S8. PANTHER overrepresentation test of PANTHER pathways for regulated porcine kidney transcripts compared to whole transcriptome, related to Figure 2

Table S9. PANTHER overrepresentation test of PANTHER Reactome pathways for regulated porcine kidney transcripts compared to whole transcriptome, related to Figure 2

Table S10. List of healthy and injured proximal tubule markers from the mouse IRI snRNA-seq dataset, along with the corresponding up- and downregulated porcine kidney transcripts, related to Figure 2

Table S11. Fisher's exact test using a 2 × 2 contingency table comparing healthy and injured proximal tubule markers from the mouse IRI snRNA-seq dataset that are present among the up- and downregulated porcine kidney transcripts, related to Figure 2 and STAR Methods

Table S12. Table of transcripts represented in the Venn diagram depicting the overlap of regulated transcripts across porcine, mouse, and human IRI datasets, related to Figure 2D

Table S13. PANTHER pathway annotation of transcripts shared among porcine, mouse, and human IRI datasets, related to Figure 2

Table S14. Mass spectrometry analysis of porcine kidney eRIC, related to Figure 3

Table S15. Proteomic analysis of human kidney eRIC samples by mass spectrometry, related to Figure 3

Table S16. PANTHER overrepresentation test showing significantly enriched GO molecular function terms among porcine RBPs identified by eRIC relative to the entire porcine proteome, related to Figure 3

Table S17. PANTHER overrepresentation test showing significantly enriched GO molecular function terms among human RBPs identified by eRIC relative to the entire human proteome, related to Figure 3

Table S18. Mass spectrometry-based proteomic analysis of whole human kidney samples, related STAR Methods

Table S19. List of RBPs included in the Venn diagram depicting shared kidney RBPs across porcine, mouse, and human datasets, related to Figure 3B

Table S20. PANTHER protein class annotation of RBPs identified in pig and human eRIC datasets, related to Figure 3

Table S21. PANTHER protein class annotation of newly identified RBPs in pig and human eRIC datasets, related to Figure 3

Table S22. List of RBPs identified in kidney eRIC that are relevant to kidney diseases, including their PANTHER protein class, related to Figure 3 The protein class of fumarylacetoacetase was manually assigned based on Gene Ontology annotation.

Table S23. List of regulated RBPs in the porcine IRI datasets, including proteome, transcriptome and eRIC data, related to Figure 4

Table S24. Table showing RBPs with differential RNA binding in porcine datasets, related to Figure 4

Table S25. Analysis of proximal tubule-specific expression of differentially RNA-binding RBPs in porcine datasets, related to Figure 4

Table S26. DNA sequence of plasmid insert, related to STAR Methods
