# Supplementary material for: RNA-binding proteome dynamics following ischemia-reperfusion injury in the kidney
Source: iScience. 2026 Nov 7;29(11):118143. doi: 10.1016/j.isci.2026.118143 (PMC13769472; doi:10.1016/j.isci.2026.118143)
Supplement: Supplementary file 1 [file mmc1.pdf]

iScience, Volume *29*

Supplemental information

RNA-binding proteome dynamics following ischemia-reperfusion injury in the kidney

Steffen Hinrichs, Katrin Bohl, Sophie Haselhorst, Francisco Lopes, Bernhard Wagener, Heike Göbel, Stefan Müller, Simon-Richard Finke, Tobias Neumann, Berit Pollmann, Constantin Rieger, Pia Rademann, Thomas Benzing, Peter Boor, Bernhard Schermer, Roman-Ulrich Müller, and Michael Ignarski

Supplemental Material

Supplemental Figures

Figure S1: Histological kidney sections from the control and IRI pig group, related to Figure 1

Figure S2: Principal component analysis of the pig kidney proteome, related to Figure 2

Figure S3: PANTHER Pathway annotations of regulated transcripts shared between porcine, mouse, and human IRI datasets, related to Figure 2

Figure S4: Principal component analysis of the porcine kidney eRIC dataset, related to Figure 3

Figure S5: Principal component analysis of the human kidney eRIC dataset, related to Figure 3

Figure S6: GO analysis of molecular function terms overrepresented in the porcine eRIC dataset, related to Figure 3

Figure S7: GO analysis of molecular function terms overrepresented in the human eRIC dataset, related to Figure 3

Figure S8: Biochemical validation of metabolic enzymes FH, EHHADH, BHMT, and SORD as RNA-binding proteins, related to Figure 4

Figure S9: Positive and negative controls for biotin-based visualization of RBP-coupled RNA, related to Figure 4

Supplementary References

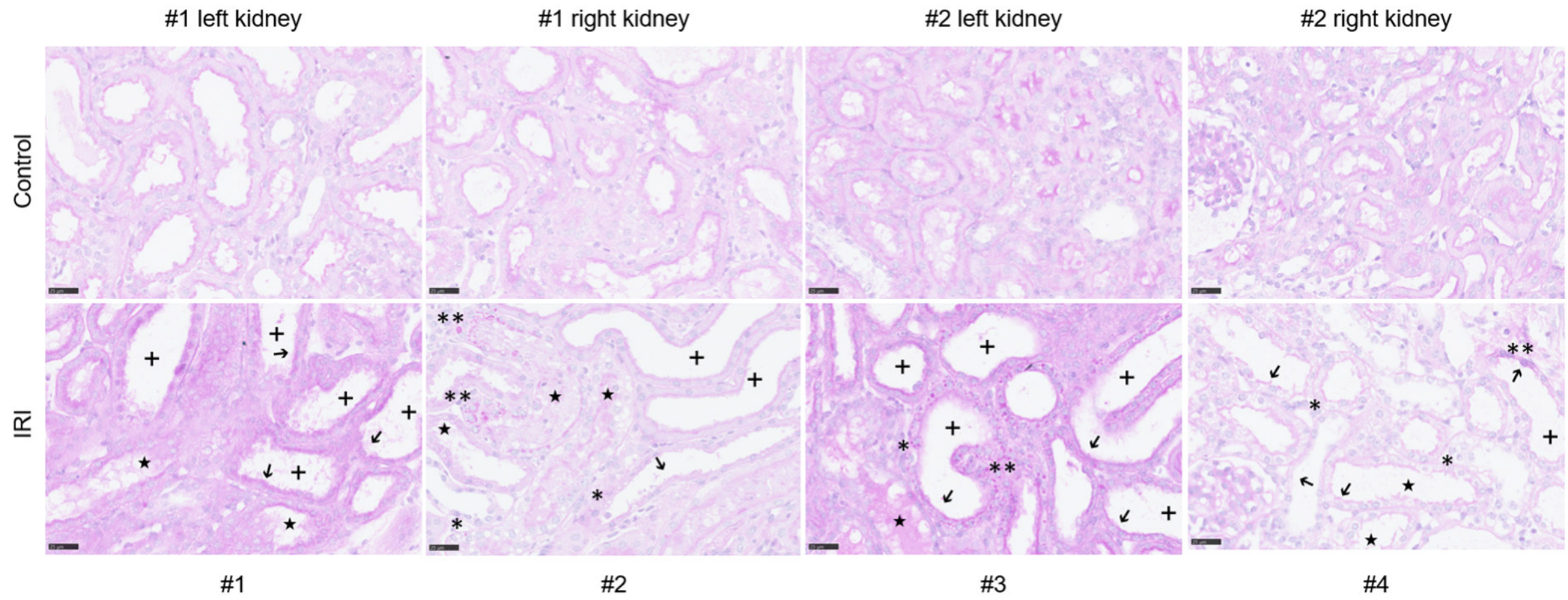

Figure S1: Histological kidney sections from the control and IRI pig group, related to Figure 1. In the IRI group, tubular dilatation (+), vacuolization with optically clear appearance (*), vacuolization with PAS-positive resorption granules (**), epithelial flattening (→) and brush border defects (★) are indicated. Sections were stained with PAS and imaged at 80x magnification. Scale bar: 25 µm

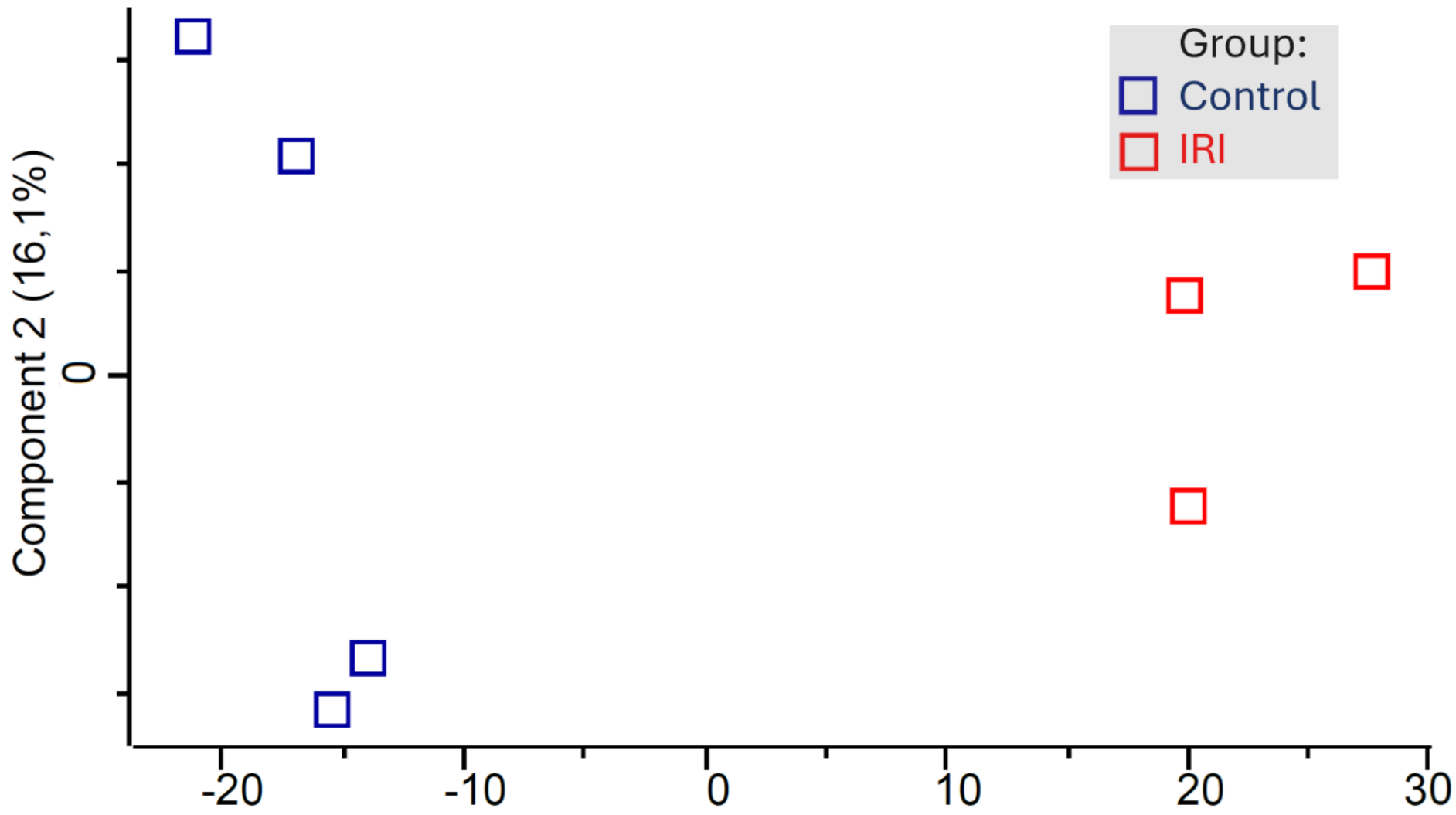

Figure S2: Principal component analysis of the pig kidney proteome, related to Figure 2. PCA based on LFQ intensities shows a clear separation along the first component between the IRI group (red, n = 3) and the control group (blue, n = 4, originating from two animals).

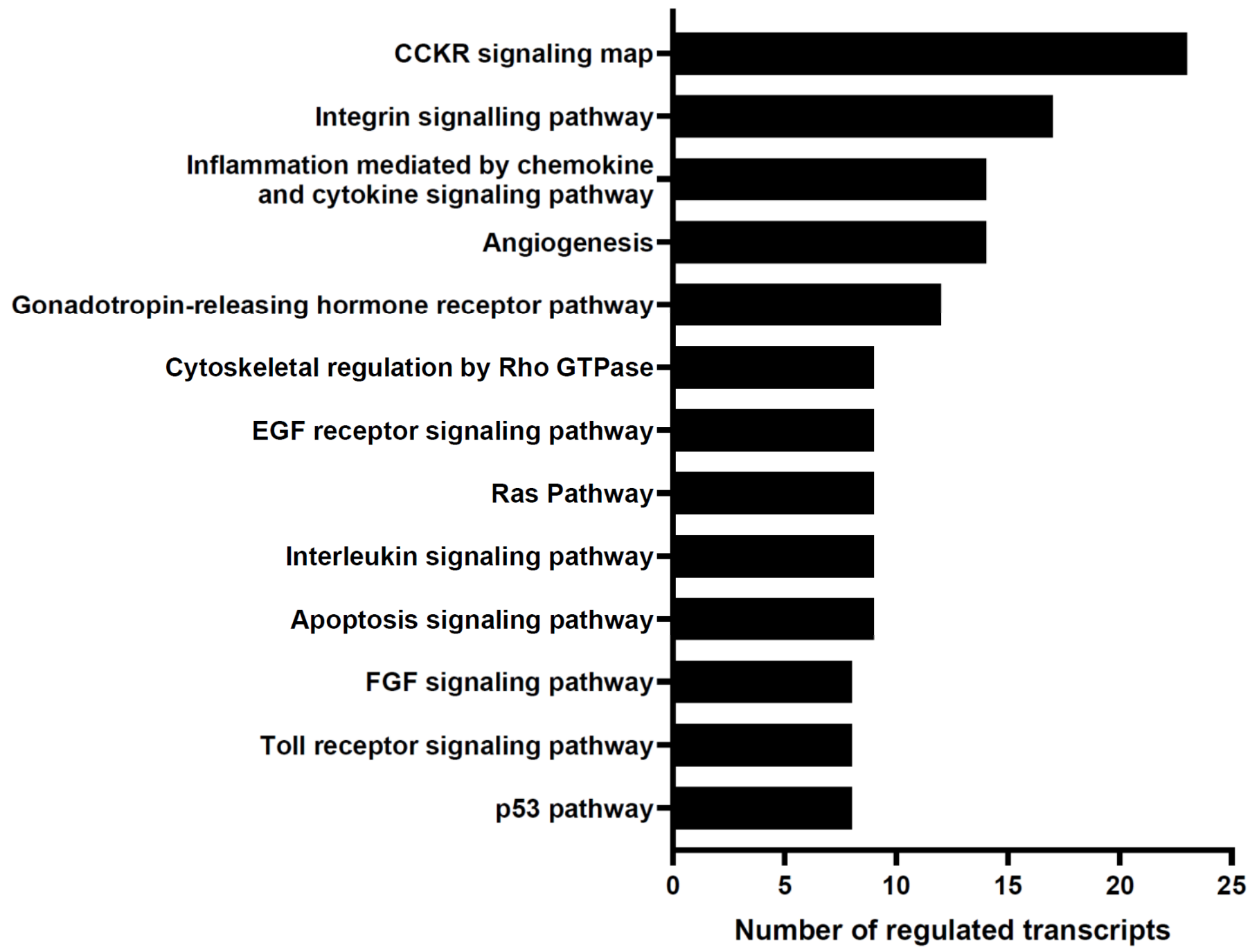

Figure S3: PANTHER Pathway annotations of regulated transcripts shared between porcine, mouse, and human IRI datasets, related to Figure 2. The bar plot displays the top 13 most abundant PANTHER Pathway annotations among transcripts commonly regulated across porcine, mouse, and human IRI datasets [S1,S2]. The y-axis indicates the specific PANTHER Pathway categories, and the x-axis shows the number of regulated transcripts associated with each pathway. Transcripts lacking PANTHER Pathway annotations are not included.

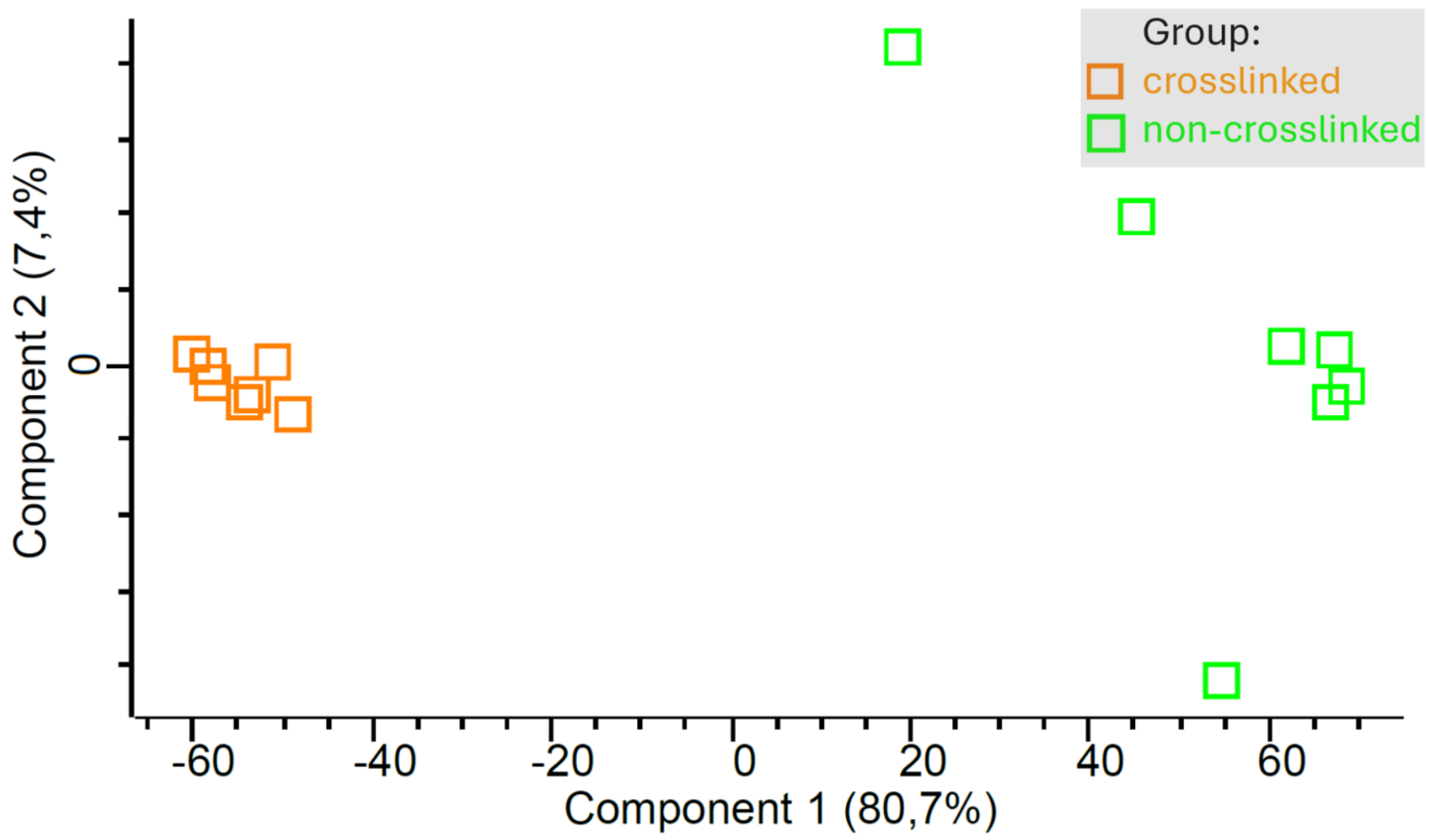

Figure S4: Principal component analysis of the porcine kidney eRIC dataset, related to Figure 3. PCA based on imputed LFQ intensities shows a clear separation along the first principal component between the crosslinked group (orange, n = 7) and the non-crosslinked group (green, n = 7).

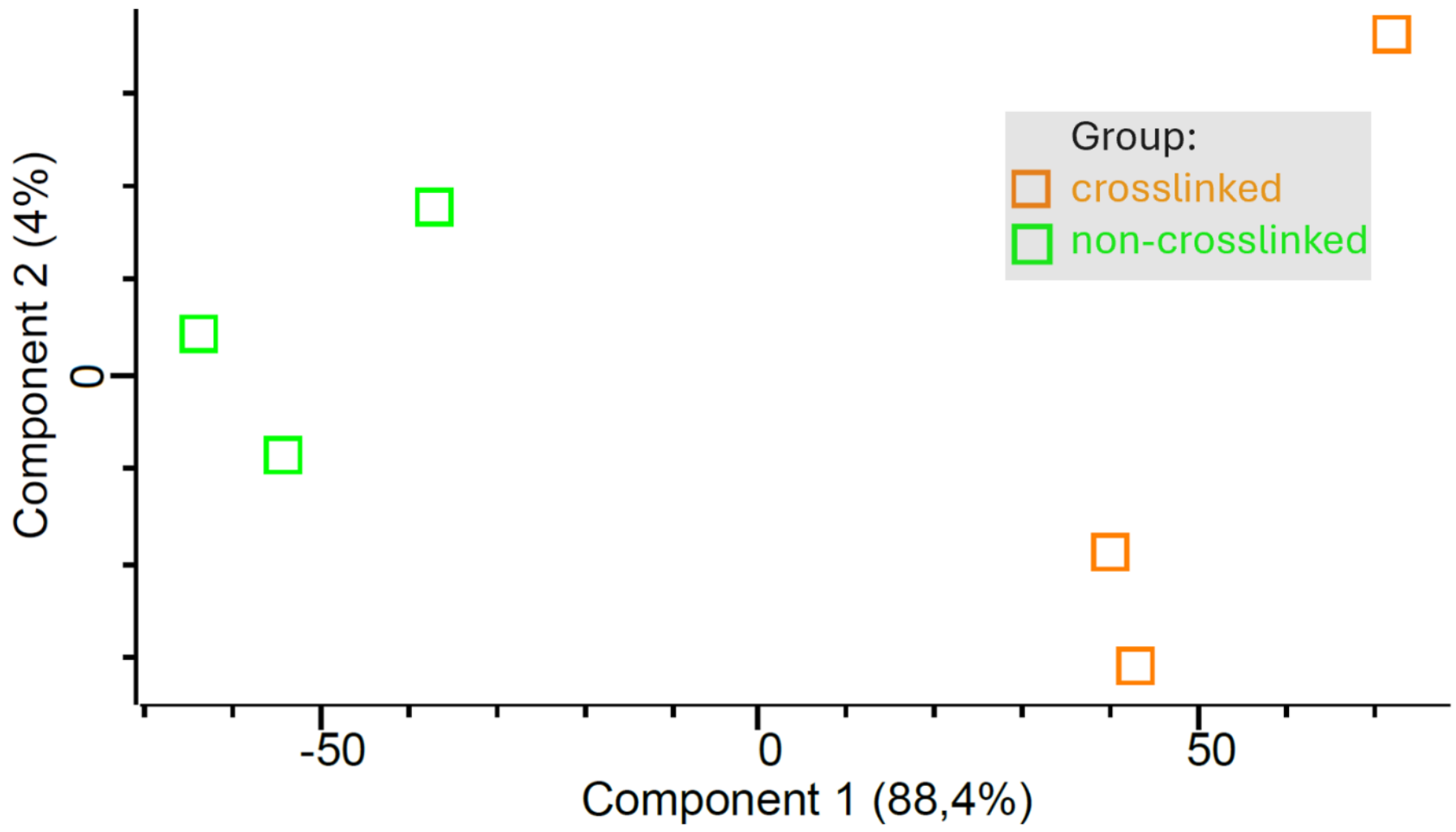

Figure S5: Principal component analysis of the human kidney eRIC dataset, related to Figure 3. PCA based on imputed LFQ intensities shows a clear separation along the first principal component between the crosslinked samples (orange, n = 3) and the non-crosslinked samples (green, n = 3).

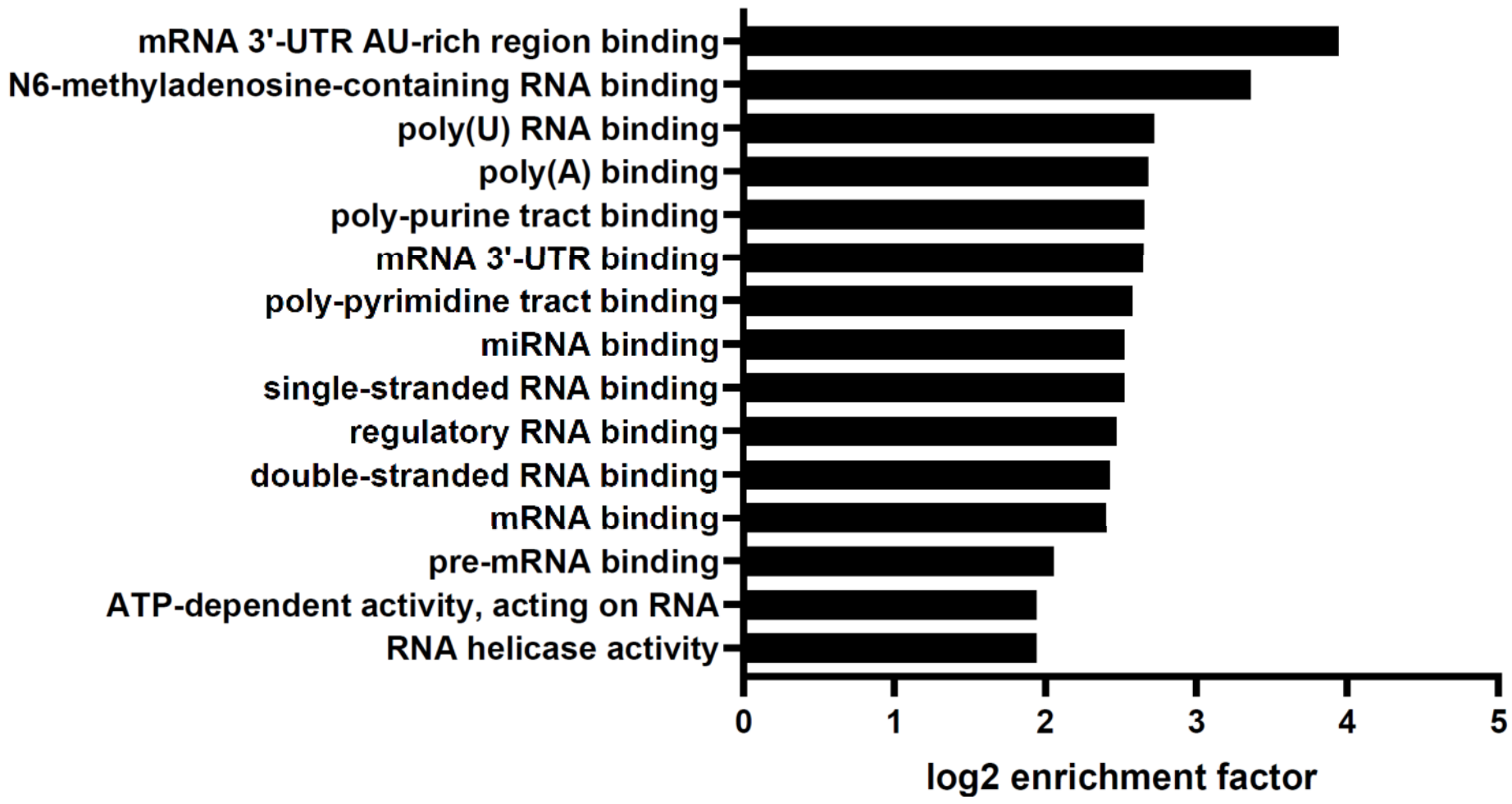

Figure S6: GO analysis of molecular function terms overrepresented in the porcine eRIC dataset, related to Figure 3. The bar plot shows significantly enriched GO molecular function (GOMF) terms identified using the PANTHER overrepresentation test, comparing gene names from the porcine eRIC dataset to the reference proteome. The x-axis represents the $\log_2$ enrichment factor.

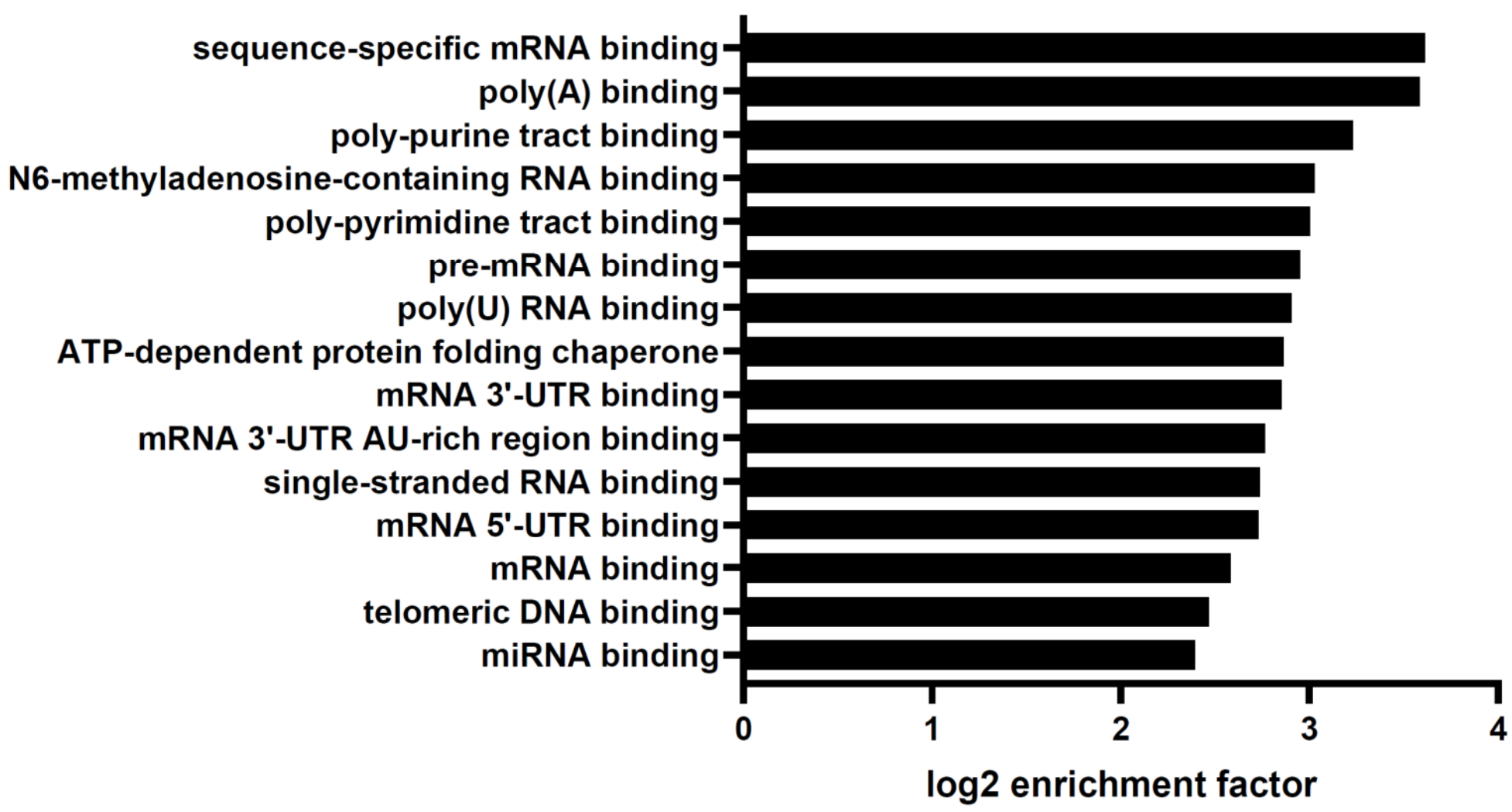

Figure S7: GO analysis of molecular function terms overrepresented in the human eRIC dataset, related to Figure 3. The Bar plot shows significantly enriched GO molecular function (GOMF) terms based on PANTHER overrepresentation test, comparing gene names from the human eRIC dataset to the reference proteome. The x-axis represents the log_2 enrichment factor.

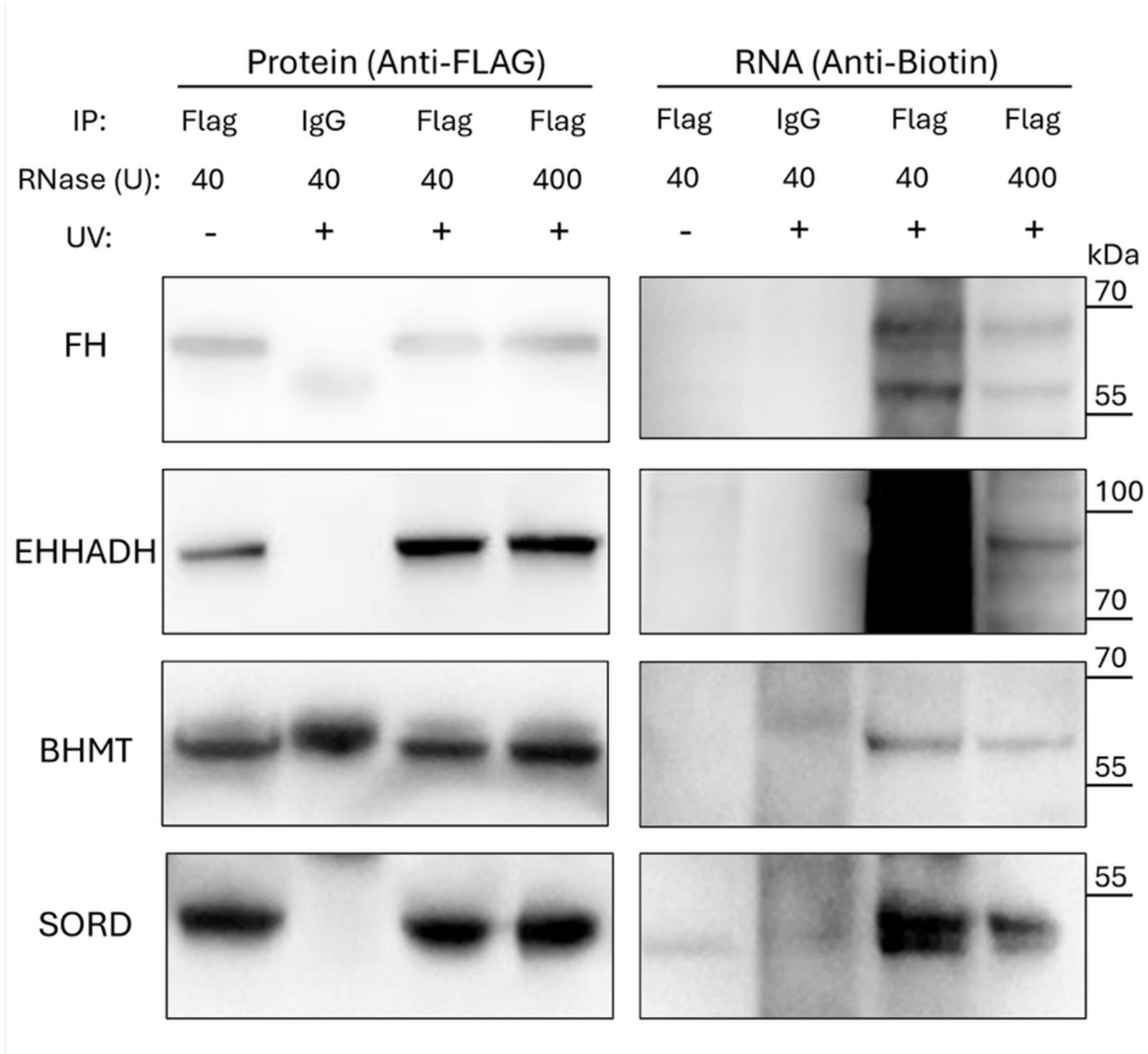

Figure S8: Biochemical validation of metabolic enzymes FH, EHHADH, BHMT, and SORD as RNA-binding proteins, related to Figure 3. Human coding sequences of FH, EHHADH, BHMT, and SORD were cloned into a 3×FLAG-pcDNA6 expression vector and transiently expressed in HEK293T cells. FLAG-tagged proteins were immunoprecipitated from UV-crosslinked (+) and non-crosslinked (−) lysates, and co-immunoprecipitated RNA was biotinylated. Crosslinked samples were treated with either 40 U or 400 U RNase I prior to analysis. Normal mouse IgG served as a negative control for immunoprecipitation specificity. Protein–RNA complexes were separated by PAGE, transferred to nitrocellulose membranes, and biotinylated RNA was detected using HRP-conjugated streptavidin. FLAG-tagged proteins were detected by immunoblotting with an anti-FLAG antibody.

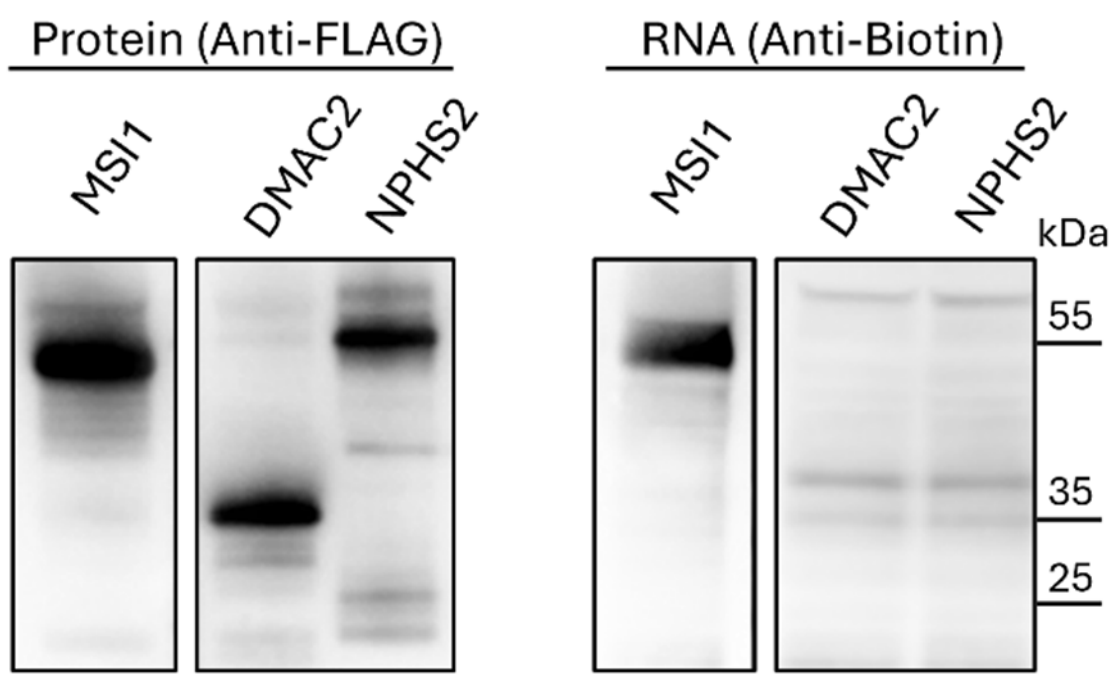

Figure S9. Positive and negative controls for biotin-based visualization of RBP-coupled RNA, related to Figure 3. FLAG-tagged MSI1 (Musashi-1) was used as a positive RBP control, whereas FLAG-tagged podocin (NPHS2) and DMAC2 were used as negative controls. Following UV crosslinking and RNase digestion, FLAG-tagged proteins were immunoprecipitated and crosslinked RNA fragments were 3′-end labeled with pCp-biotin and detected by streptavidin blotting. Protein expression and immunoprecipitation were confirmed by anti-FLAG immunoblotting. A clear RNA-associated biotin signal was detected for MSI1, whereas no corresponding signal was detected at the expected molecular weight of podocin (NPHS2) or DMAC2.

Supplemental References

[S1] Hinze C., Kocks C., Leiz J., Karaiskos N., Boltengagen A., Cao S., Skopnik C.M., Klocke J., Hardenberg J.H., Stockmann H., et al (2022). Single-cell transcriptomics reveals common epithelial response patterns in human acute kidney injury. Genome Med.14(1):103. doi:10.1186/s13073-022-01108-9
[S2] Johnsen M., Kubacki T., Yeroslaviz A., Späth M.R., Mörsdorf J., Göbel H., Bohl K., Ignarski M., Meharg C., Habermann B., et al (2020). The Integrated RNA Landscape of Renal Preconditioning against Ischemia-Reperfusion Injury. J Am Soc Nephrol.31(4):716-730. doi:10.1681/asn.2019050534
